# Target gene expression levels and competition between transfected and endogenous microRNAs are strong confounding factors in microRNA high-throughput experiments

**DOI:** 10.1186/1758-907X-3-3

**Published:** 2012-02-10

**Authors:** Takaya Saito, Pål Sætrom

**Affiliations:** 1 Department of Cancer Research and Molecular Medicine, Norwegian University of Science and Technology, Prinsesse Kristinsgt. 1, NO-7491 Trondheim, Norway; 2 Department of Computer and Information Science, Norwegian University of Science and Technology, Sem Sælands vei 9, NO-7491 Trondheim, Norway

**Keywords:** microRNA targets, siRNA, microarray, proteomics, PAR-CLIP

## Abstract

**Background:**

MicroRNA (miRNA) target genes tend to have relatively long and conserved 3' untranslated regions (UTRs), but to what degree these characteristics contribute to miRNA targeting is poorly understood. Different high-throughput experiments have, for example, shown that miRNAs preferentially regulate genes with both short and long 3' UTRs and that target site conservation is both important and irrelevant for miRNA targeting.

**Results:**

We have analyzed several gene context-dependent features, including 3' UTR length, 3' UTR conservation, and messenger RNA (mRNA) expression levels, reported to have conflicting influence on miRNA regulation. By taking into account confounding factors such as technology-dependent experimental bias and competition between transfected and endogenous miRNAs, we show that two factors - target gene expression and competition - could explain most of the previously reported experimental differences. Moreover, we find that these and other target site-independent features explain about the same amount of variation in target gene expression as the target site-dependent features included in the TargetScan model.

**Conclusions:**

Our results show that it is important to consider confounding factors when interpreting miRNA high throughput experiments and urge special caution when using microarray data to compare average regulatory effects between groups of genes that have different average gene expression levels.

## Background

MicroRNAs (miRNAs) are an abundant class of small non-coding RNAs (ncRNAs) that negatively regulate protein-coding genes [1,2]. MicroRNAs are involved in many important regulatory roles [3-5], and current estimates indicate that miRNAs regulate at least 60% of the human protein-coding genes [6].

In animals, functional miRNA sites preferentially reside in 3' UTRs [7], and these sites are generally well conserved [6]. Moreover, some ubiquitously expressed genes, such as housekeeping genes, have shorter 3' UTRs to potentially avoid miRNA regulation [2,8], whereas proliferating cells express mRNAs with shortened 3' UTRs to avoid miRNA regulation [9]. Hence, miRNA target genes are likely to have relatively long and conserved 3' UTRs. However, to what degree the length and conservation of 3' UTR contribute to miRNA targeting is still poorly understood. To illustrate, data from Argonaute RNA immunoprecipitation (RIP) in human and fly indicate that miRNAs target short 3' UTRs [10,11], whereas microarray data from miRNA transfection experiments and sequence data from Argonaute cross-linked immunoprecipitation (CLIP) experiments indicate that miRNAs target long 3' UTRs [12]. Wen and colleagues also found that target site conservation was more important for CLIP-supported target sites than for targets that were down-regulated in the transfection experiments [12]. Moreover, our previous study [13] showed that genes with a 3' UTR longer than 4,000 nucleotides were less affected by ectopically expressed miRNAs than were genes with a shorter 3' UTR, and that target site conservation had little or no effect on the performance of our miRNA target prediction algorithm.

In addition to 3' UTR length and conservation, several other gene characteristics also affect miRNA regulation. For example, many miRNAs are known to regulate genes involved in cell development processes [3]. Another example is that miRNAs appear to preferentially target genes with high CpG promoters [14]. Also, as highly expressed genes transcribe a large number of mRNAs, the miRNA regulation of those mRNAs can be different from those of weakly expressed genes, although current analyses disagree on whether miRNAs affect highly expressed genes more or less than medium or lowly expressed genes [15,16].

High throughput experiments based on microarrays or proteomics have been important for characterizing miRNA regulation [17-20]. Although these and more recent comparative studies [10,12] found that some features such as seed complementarity and seed strength are consistently important for miRNA targeting, other features such as 3' UTR length and site conservation mentioned above differ between studies and technologies. There are at least three potential features that may contribute to the difference between miRNA high-throughput experiments. First, the number of genes that cover a microarray experiment is usually much larger than that of a proteomics experiment. For instance, Baek *et al*. [20] used both microarray and proteomics for their miRNA target gene analysis, and the number of genes detected for microarray and proteomics samples were about 20,000 and 2,000, respectively. Second, transfected (exogenous) miRNAs compete with endogenous miRNAs for the protein complex needed for miRNA regulation [21]. Therefore, genes targeted by endogenous miRNAs but not by the exogenous miRNA can be up-regulated. Third, the effect of miRNA regulation can be diluted by target abundance, which means that each target gene is less down-regulated when the miRNA has many highly expressed compared with a few lowly expressed target genes [22]. Common for these features is that they are target site-independent, but gene context-dependent.

In this study, we have investigated the effects on miRNA targeting of several such target site-independent but gene context-dependent features. We categorized these features into three types: (i) target mRNA features, such as 3' UTR length, 3' UTR sequence conservation, and mRNA expression level; (ii) sample features, such as the competition and dilution effects; and (iii) platform features, such as different types of technologies and experimental methods. We found that two features - the competition effect between endogenous miRNAs and transfected miRNAs, and mRNA expression level - have a strong impact on results from high throughput experiments. Both features are confounding factors that explain many of the previously reported differences between different studies and high throughput technologies. It is important to consider these confounding factors in order to analyze accurately and robustly different types of miRNA high-throughput experiments and to infer correctly the characteristics of miRNA regulation.

## Results and discussion

### Target mRNA features: ectopic miRNA expression differentially affects subgroups of genes with differing 3' UTR length, 3' UTR conservation, and mRNA expression level

As we expected that mRNAs targeted by miRNA have long and conserved 3' UTRs, we wanted to examine how these characteristics actually affect miRNA regulation. Specifically, we wondered whether there was a difference in how different gene groups, such as genes with long, medium, or short 3' UTRs or genes with high, medium, or low 3' UTR conservation, were affected by ectopic miRNA expression. To address this question, we used microarray and proteomics data from five and two miRNA transfection experiments, respectively, and microarray data from two miRNA inhibition experiments and analyzed the differences in gene expression log ratio values of predicted targets in the different gene groups (see Methods). We also included microarray data from a small interfering RNA (siRNA) transfection experiment because siRNAs behave as miRNAs in terms of target recognition [23,24]. In contrast to evolutionary selected miRNA targets, however, targets for artificially designed exogenous siRNAs should be evolutionary unbiased. The siRNA dataset, therefore, served as an estimate of the general regulatory effects of over-expressing small RNAs. In total, we used 10 different types of miRNA high-throughput experiments, which covered 140 samples and 70 miRNAs and siRNAs (Additional file 1, Table S1 and S2).

**Figure 1:**
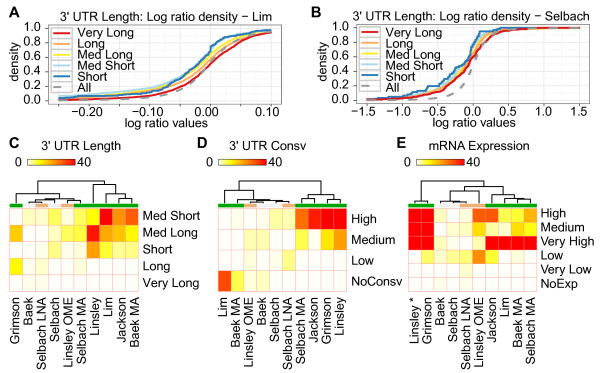
**Heatmaps show effects of ectopic miRNA regulation for sub-groups of 3' UTR length, 3' UTR conservation, and mRNA expression level**. Three heat maps show -log (base 2) transformed *P*-values for (**C**) 3' UTR length, (**D**) 3' UTR conservation, and (**E**) mRNA expression. We added two cumulative density plots for Lim (**A**) and Selbach (**B**) to illustrate the multiple non-parametric tests for the sub-groups of 3' UTR lengths; 'All' is the cumulative density for all the genes measured in the experiment and represents the reference distribution. 'All' genes include both predicted miRNA target and non-target genes. Although the mRNA expression data (E) was cell type specific and for HeLa cells, we included the Linsley dataset (from HCT116 and DLD-1 colon tumor cells) in the heat map for comparison purpose (indicated with '*'). The color labels under the dendrogram represent green for microarray of transfection assay, gray for proteomics of transfection assay, and orange for both microarray and proteomics with inhibition assay. miRNA, microRNA; mRNA, messenger RNA; UTR, untranslated region.

For each miRNA and siRNA, we first separated predicted miRNA or siRNA target genes from the rest. The predicted target genes were genes that have at least one canonical seed site in their 3' UTRs (see Methods). We used the set of predicted miRNA or siRNA target genes to analyze miRNA down-regulation effects on three different target mRNA features: (i) 3' UTR length, (ii) 3' UTR conservation, and (iii) mRNA expression level as determined by RNA-Seq counts. We split these three features into smaller sub-groups as described in the Methods section. We then used a one-sided Wilcoxon rank-sum test to determine whether the predicted target genes in one sub-group were significantly more down-regulated than the genes in the rest of the sub-groups (Figure 1). To illustrate, Figures 1A and 1B show the cumulative density plots of the log-ratio values for the 3' UTR length sub-groups on the Lim microarray and Selbach proteomics datasets [17,19]. In the Lim dataset (Figure 1A), the sub-group Med Short, representing genes with 3' UTRs in the range of 248 to 629 nucleotides (nts), was significantly left-shifted and, therefore, more down-regulated than the rest as indicated in the corresponding heatmap (Figure 1C; *P*-value 4.02e-17; Additional file 1, Table S3). In the Selbach proteomics dataset (Figure 1B), the Short sub-group was most shifted to the left compared to the others, but the differences were not as significant as in the Lim dataset - likely due to the smaller dataset (Figure 1C; *P*-value 0.02; Additional file 1, Table S3). Additional file 1, Tables S3 to S5 summarize all *P*-values of the one-sided Wilcoxon rank-sum test on 3' UTR length, 3' UTR conservation, and mRNA expression; Kolmogorov-Smirnov tests on the same sub-groups gave similar results (Additional file 1, Tables S6 to S8). The following sections describe and discuss the results for each target mRNA feature.

### Target mRNA features: predicted target genes with short 3' UTRs are more down-regulated than genes with long 3' UTRs

We have previously reported that genes with very long 3' UTRs (> 4,000 nts) are poor targets for ectopically expressed miRNAs or siRNAs [13]. Consistent with this, there was no experiment that showed significant down-regulation for the Very Long sub-group (Figure 1C, Additional file 1, Table S3). Interestingly, genes with short or medium 3' UTRs (Short, Med Short, and Med Long) were significantly down-regulated compared to genes with long 3' UTRs (Long and Very Long) among nearly all of the experiments. We saw the strongest effect for 3' UTRs with sub-groups Med Short and Med Long, as one or both groups were significantly down-regulated compared to other predicted targets in eight of ten experiments (Additional file 1, Table S3). Between these two sub-groups, Med Short had lower *P*-values than Med Long in seven of ten experiments. Using the one-sided Kolmogorov-Smirnov test as an alternative test method also supported the same trends (Additional file 1, Table S6). These results indicate that miRNA or siRNA target genes with short 3' UTRs were generally more down-regulated than genes with long 3' UTRs.

### Target mRNA features: conservation in 3' UTR regions has inconsistent regulatory effects

Although the sub-group of highly conserved 3' UTR regions was strongly down-regulated in five of ten experiments, the non-conserved sub-group was down-regulated in two of ten experiments (Figure 1D; Additional file 1, Table S4). One possible explanation for this inconsistent pattern could be the conservation levels of the miRNAs used in the experiments. Since highly conserved miRNAs tend to have more target genes than less-conserved miRNA, highly conserved miRNAs may be more affected by the reported target dilution effect [22]. However, we found no significant correlations between the degree of miRNA conservation and the down-regulation effects (data not shown). Moreover, the siRNAs used in the Jackson experiment should be unaffected by 3' UTR conservation, but these siRNAs strongly down-regulated highly conserved 3' UTRs compared with other 3' UTRs [24]. We observed the same inconsistency for 3' UTR conservation when testing with the one-sided Kolmogorov-Smirnov test (Additional file 1, Table S7). Thus, although highly conserved 3' UTRs in some cases can be better targets for ectopically expressed small RNAs, the inconsistent regulatory effects suggest that other factors are more important.

### Target mRNA features: predicted target genes with high mRNA expression levels are more down-regulated than the genes with low mRNA expression levels

A recent study showed that mRNA expression affects siRNA efficacy such that lowly expressed mRNAs are less affected by siRNAs than are highly expressed mRNAs [15]. Consistent with these results, siRNA and miRNA target genes with high or medium expression as measured by RNA-seq [25] were significantly down-regulated compared to the rest of the sub-groups in the microarray experiments (Figure 1E). We saw a very strong down-regulatory effect on mRNA expression with sub-groups Very High, High, and Medium, as one or more sub-groups were significantly down-regulated compared to other predicted targets in seven of ten experiments (Additional file 1, Table S5). Moreover, there was no experiment that showed significant *P*-values for sub-groups Very Low and NoExp. One-sided Kolmogorov-Smirnov tests also gave similar results (Additional file 1, Table S8). These results indicate that predicted miRNA or siRNA target genes with high or moderate expression levels are generally more down-regulated than genes with low expression levels.

### Target mRNA features: comparison tests on individual samples strongly support that the differences between subgroups are common for many miRNAs

Our analyses so far showed clear differences in how ectopic miRNA and siRNA expression affected certain subgroups of genes. Since these differences were based on the average effects of multiple miRNAs, however, we could not exclude that these differences were due to a few miRNAs instead of being common effects for many miRNAs. To test this possibility, we repeated the tests of the three target mRNA features - 3' UTR length, 3' UTR conservation, and mRNA expression levels - individually on the 140 different samples (Additional file 1, Table S2). We then calculated the proportions of samples that showed significant *P*-values (Additional file 1, Tables S9-S11) and defined these proportions as Sample level scores (see Methods). Consequently, a subgroup with Sample level score = 0.5 would be significant in 70 (50%) of the individual experiments. To compare the trends of the test results between experiments and samples, we created two types of counts to represent the trends for both experiments and samples: (i) the number of experiments that had significant *P*-values from the test results of the experiments, and (ii) the number of experiments that had their Sample level scores greater than 0.5 (Table 1). The trends of mRNA expression level for experiments and samples were very similar for (Pearson r = 0.91; *P*-value = 0.01), whereas the trends of two other features showed high but insignificant correlation coefficients (3' UTR length, r = 0.79, *P*-value = 0.11; 3' UTR conservation, r = 0.81, *P*-value = 0.19). The results show that many individual samples support the overall trends for each experiment set, especially for the mRNA expression level features.

**Table 1:** Multiple comparison tests at an individual sample level support the experimental level test results

Factor	Subgroup	Expr	Smpl
3' UTR Length	Very Long	0	0
	Long	1	0
	Med Long	7	3
	Med Short	6	3
	Short	5	0
3' UTR Consv	High	5	3
	Medium	3	1
	Low	1	1
	NoConsv	2	0
mRNA Exp	Very High	6	6
	High	7	5
	Medium	5	4
	Low	5	2
	Very Low	0	0
	NoExpr	0	0

Consv, conservation; Expr, the number of experiments that have significant *P*-value; Smpl, the number of experiments that have Sample level scores greater than 0.5; UTR, untranslated region.

### Sample features: competition with endogenous miRNAs impacts exogenous miRNAs' targeting of genes with long 3' UTRs

Small RNA transfection perturbs endogenous miRNA regulation such that genes targeted by endogenous miRNAs can become up-regulated [21]. We, therefore, hypothesized that genes with long 3' UTRs had a net unaffected expression or reduced response because these genes potentially had more endogenous miRNA target sites than genes with short 3' UTRs. To test this hypothesis, we separated the genes into four groups based on whether the genes were predicted to be targeted by the exogenous miRNAs and by highly expressed endogenous miRNAs. Both the first (T +Endo) and the second (T -Endo) groups consist of genes targeted by exogenous miRNAs, whereas the third (NT +Endo) and the fourth (NT -Endo) groups consist of genes without exogenous miRNA targets. The second word in the group names indicates that the group contains either genes targeted by endogenous miRNAs (+Endo) or genes without endogenous miRNA targets (-Endo) (Additional file 1, Table S12; see Methods). Specifically, we used two of the four groups, T -Endo and T +Endo, for statistical analysis. We only used eight transfected experiments and excluded two inhibition experiments because Selbach locked nucleic acid (LNA) and Linsley 2'-O-methyl (OME) experiments inhibited endogenous miRNAs.

**Figure 2:**
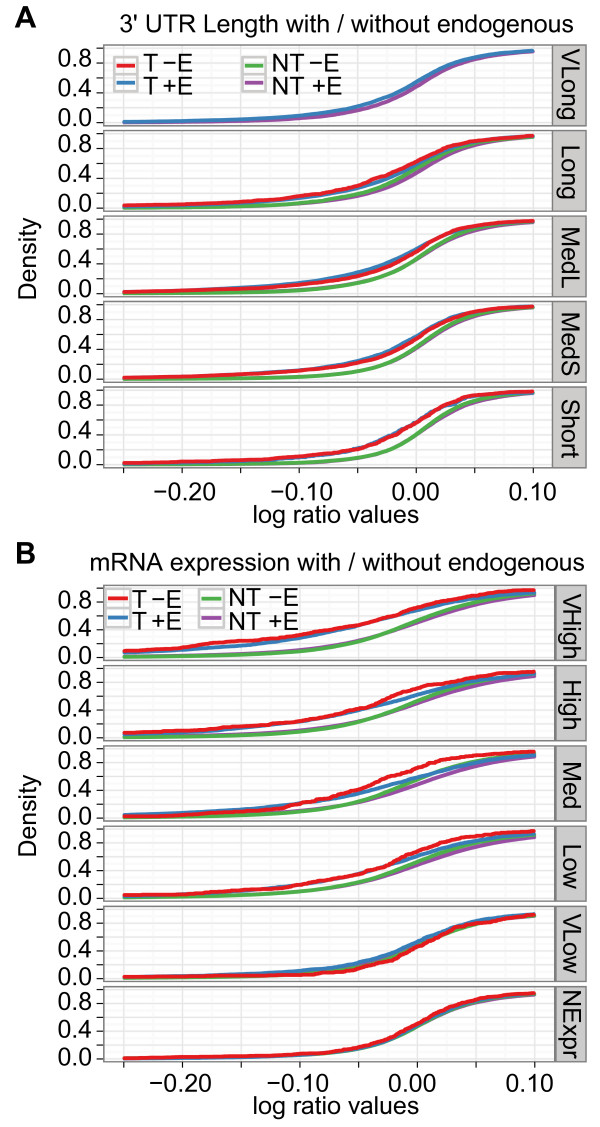
**Endogenous miRNAs tend to target genes with long 3' UTRs and exogenous miRNAs target highly expressed genes that had a small influence of endogenous miRNAs**. Two cumulative density plots of the log-ratio values show the miRNA down-regulatory effects on sub-groups of (**A**) 3' UTR length with the Grimson dataset and (**B**) mRNA expression level with the Jackson dataset for ectopically expressed miRNA or siRNA target genes that were separated into T +Endo (T +E), T -Endo (T -E), NT +Endo (NT + E), and NT -Endo (NT -E). miRNA, microRNA; mRNA, messenger RNA; siRNA, small interfering RNA; UTR, untranslated region.

Consistent with our hypothesis, there were no T -Endo genes that belonged to the Very Long sub-group in any of the eight experiments (Figure 2A; Additional file 1, Table S13). For the other sub-groups of 3' UTR length, we tested whether T -Endo genes were more down-regulated than T +Endo genes (Additional file 1, Table S13). Although the majority of experiments showed no significant differences for the subgroups, T -Endo genes were significantly more down-regulated than T +Endo genes for the sub-group Long in three of eight experiments. Moreover, the only other significant difference was for the Med Long sub-group on the Selbach dataset, where T -Endo genes again were significantly more down-regulated than T +Endo genes. These results suggest that genes with a very long 3' UTR are less affected by exogenous miRNAs than are other genes because these genes have a higher chance of being under the influence of endogenous miRNAs.

### Sample features: PAR-CLIP data show that endogenous miRNAs target most mRNAs with long 3' UTR

To further test whether endogenous miRNAs target most mRNAs with very long 3' UTR, we analyzed the data from an experiment that used Photoactivatable-Ribonucleoside-Enhanced Crosslinking and Immunoprecipitation (PAR-CLIP) of the four human Argonautes (AGO1-4, also known as EIF2C1-4) to identify miRNA binding sites [16]. We mapped PAR-CLIP-supported AGO binding sites in 3' UTR regions and for each of the five different sub-groups defined by 3' UTR length, we counted the number of genes with binding sites (Table 2). We found more AGO binding sites in long 3' UTR genes (Very Long and Long) than in short 3' UTR genes (Med Long, Med Short, and Short) when compared with all available RefSeq genes (*P*-values < 2.2e-16, Fisher's exact test for all four AGOs). Specifically, of the five subgroups, the genes with very long 3' UTRs (Very Long) had the highest fraction of genes with AGO binding sites and this fraction decreased with decreasing 3' UTR length (Table 2). These results support that endogenous miRNAs preferentially target mRNAs with very long 3' UTR.

**Table 2:** PAR-CLIP data show that endogenous miRNA tend to target mRNAs with long 3' UTR

	Very Long	Long	Med Long	Med Short	Short
AllRef	917	6194	6201	6198	6201
AGO1	787 (85.82%)	3757 (60.66%)	2371 (38.24%)	1123 (18.12%)	471 (7.60%)
AGO2	320 (34.90%)	1010 (16.31%)	428 (6.90%)	148 (2.39%)	64 (1.03%)
AGO3	883 (96.29%)	4800 (77.49%)	3332 (53.73%)	1804 (29.11%)	639 (10.30%)
AGO4	713 (77.75%)	3087 (49.84%)	1510 (24.35%)	639 (10.31%)	167 (2.69%)

The table shows the total number of genes (AllRef) and the number of Argonaute-bound 3' UTRs (AGO1-4) for each sub-group defined by 3' UTR length (see Methods). Values in brackets indicate the percentage of all genes in the sub-group that have AGO binding sites. miRNA, microRNA; mRNA, messenger RNA; PAR-CLIP, Photoactivatable-Ribonucleoside-Enhanced Crosslinking and Immunoprecipitation; UTR, untranslated region.

### Sample features: competition with endogenous miRNAs is not affected by evolutionary conservation levels of 3' UTRs

The test results of mRNA target features showed no consistent evidence that genes in any sub-group of 3' UTR conservation were significantly more down-regulated than were the genes in the rest of the sub-groups (Additional file 1, Table S4). We, therefore, did not expect to find consistent patterns of interaction between the competition effect and 3' UTR conservation. Indeed, when we tested whether T -Endo genes were more down-regulated than T +Endo genes in the sub-groups of 3' UTR conservation, the test showed no consistent trends across sub-groups; rather, the tests indicated consistent differences between experiments, as all sub-groups were significant on the Lim, Jackson, and Selbach datasets (Additional file 1, Table S14). These results suggest that competition between endogenous and exogenous miRNAs is unaffected by the levels of evolutionary conservation on 3' UTRs.

### Sample features: competition with endogenous miRNAs has a strong impact on genes with medium or higher mRNA expression levels

As endogenous miRNA regulation mostly reduces target mRNA expression [8,26,27], miRNA target genes with low mRNA expression levels would more likely be under strong regulation by endogenous miRNAs. Similarly, miRNA target genes with high mRNA expression would less likely be under strong endogenous miRNA regulation. Consequently, we expected lowly expressed mRNAs to be less affected by competition with exogenous miRNAs than were highly expressed mRNAs. Indeed, T -Endo genes were significantly more down-regulated than were T +Endo genes for high or moderate mRNA expression levels (Very High, High, and Medium; Figure 2B; Additional file 1, Table S15). Moreover, no experiments showed significant down-regulation for low or no mRNA expression levels (Very Low and NoExp). As, according to our results, endogenous miRNAs preferentially target genes with long 3' UTRs, taken together, these results support that the genes with a very long 3' UTR are less affected by exogenous miRNAs because most of them are under the influence of endogenous miRNAs.

### Sample features: tests on individual samples support that the competition effect is strongest for genes with strong mRNA expression levels

To analyze further the trends of competition effects on 3' UTR length, 3' UTR conservation, and mRNA expression level, we tested the difference of miRNA down-regulation between T -Endo and T +Endo genes on the 140 individual samples instead of the collective experiments. To compare the trends of the test results between experiments and samples, we again created two types of counts: (i) the number of experiments that had significant *P*-values from the test results of the experiments, and (ii) the number of experiments that had their Sample level scores greater than either 0.5 or 0 (Additional file 1, Table S16). With a strict threshold of Sample level scores (> 0.5), the tests on samples showed no strong support for the trend observed when tested on the experiments. With a less strict threshold of Sample level scores (> 0), the tests on samples supported the trend for mRNA expression level (Pearson r = 0.86; *P*-value = 0.03), whereas the 3' UTR length showed some, but insignificant, support of the experiment-level results (r = 0.47; *P*-value = 0.42). The 3' UTR conservation showed no correlation with the experiment-level results (r = -0.10; *P*-value = 0.9). Together, the results indicated that some samples support the result from the collective experiments that the competition effect more strongly affects genes with high compared with low mRNA expression level.

### Platform features: microarray datasets can have cryptic bias towards detecting differential expression in highly expressed genes

A recent study showed that mRNA expression affects siRNA efficacy such that lowly expressed mRNAs are less affected by siRNAs than are highly expressed mRNAs [15]. Consistent with these results, siRNA and miRNA target genes with high or medium expression, as measured by RNA-Seq [25], were significantly down-regulated compared to the rest of the sub-groups in the microarray experiments (Figure 1E). However, these trends were not apparent in the proteomics datasets, which instead showed significant effects on the lowly expressed genes (two of three experiments; Additional file 1, Table S5).

**Figure 3:**
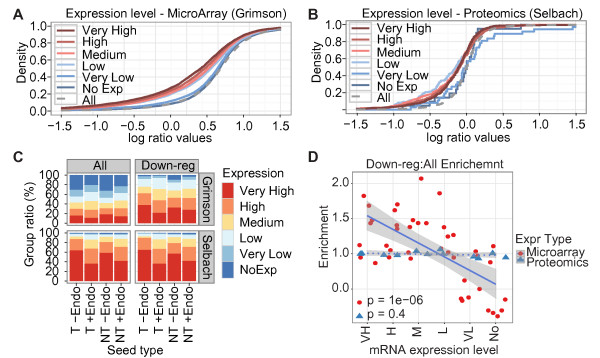
**Microarrays but not proteomics are biased towards detecting differential expression among highly expressed genes**. Cumulative density plots of log-ratio values for miRNA targets grouped by gene expression levels in (**A**) the Grimson and (**B**) the Selbach datasets. (**C**) Barplots show the ratio of the six sub-groups of mRNA expression levels subdivided by predicted exogenous and endogenous miRNA targeting in the Grimson and Selbach datasets for all genes ('All') and down-regulated genes ('Down-reg' *P *< 0.01; log ratio (lr) < -0.01). (**D**) Scatter plots show log_2 _enrichment of down-regulated genes compared with all genes for the six sub-groups of mRNA expression levels in all studied datasets. Lines and shaded grays show respectively linear fits and standard errors for the microarray (red dots) and proteomics (blue triangles) experiments; *P*-values (lower left) are unadjusted *P*-values from Pearson correlation tests. Data points based on a single gene were excluded. The regression lines show that in the microarray but not the proteomics experiments, down-regulated genes are enriched among highly expressed genes and that this enrichment depends on gene expression levels. miRNA, microRNA; mRNA, messenger RNA.

The cumulative density plots of log-ratio values for the Grimson microarray and Selbach proteomics datasets illustrate the differences (Figure 3A, B). The three sub-groups of very high, high, and medium expression were left-shifted and, therefore, more strongly down-regulated relative to the other groups in the Grimson microarray dataset (Figure 3A). Indeed, the groups' expression level appeared to strictly determine the degree of down-regulation, as the very highly expressed genes were more left-shifted compared to the highly expressed genes and so on. In contrast, the Selbach proteomics datasets showed no such trends (Figure 3B); the three sub-groups with the highest expression levels were similarly affected, whereas the lowly expressed genes were slightly more down-regulated than the other groups.

As the proteomics experiments relied on detecting and identifying individual proteins, whereas microarrays use hybridization signals to infer gene expression, we reasoned that the differences might be explained by differences in the sensitivity of the two methods to detect highly and lowly expressed genes or to detect expression changes for highly and lowly expressed genes. Whereas the microarray signals were evenly distributed among all subgroups of gene expression levels, the proteomics data showed bias towards highly expressed genes and detected few very lowly expressed genes (Figure 3C). These trends were apparent in the other microarray and proteomics datasets as well (Additional file 1, Figure S1). When considering expression changes, however, the proteomics data showed little bias and detected down-regulated genes independently of their expression level (Figure 3D). The microarray data, in contrast, showed strong expression-related bias, such that down-regulated genes were enriched among the highly expressed genes and depleted among the lowly expressed genes.

Importantly, these results were independent of miRNA targeting, as subdividing the genes into four groups based on whether the genes contained predicted target sites for the exogenous miRNAs and highly expressed endogenous miRNAs gave similar results (Additional file 1, Figure S2). This grouping further illustrated the effects of miRNA targeting, however. Specifically, consistent with miRNAs inhibiting mRNA expression, the most highly expressed genes constituted a smaller percentage of the genes predicted to be targets for endogenous miRNA (T +Endo and NT +Endo, Figure 3C) than of the genes predicted not to be targets (T -Endo and NT -Endo; Figure 3C).

In summary, the exogenous miRNAs' apparent strong effects on highly expressed genes within the microarray data can be explained by technology-related artifacts. Specifically, our results show that although microarrays detect lowly expressed genes, arrays have lower sensitivity for identifying differential expression for such genes than for highly expressed genes. This is consistent with previous results [28]. Proteomics data, in contrast, are biased towards highly expressed genes, but detect differential expression independently of gene expression levels. In other words, proteomics fails to detect many genes with a low expression level but the sensitivity in detecting differentially expressed genes is similar among different expression levels, whereas microarrays can detect genes with a low expression level but the sensitivity in detecting differential expression is low for these genes compared with highly expressed genes. Because of these differences, microarray but not proteomics data will show that miRNAs on average have a stronger effect on highly than on lowly expressed genes.

### Platform and Sample features: competition with endogenous miRNAs has a stronger impact on regulation than has dilution from high overall target expression

**Figure 4:**
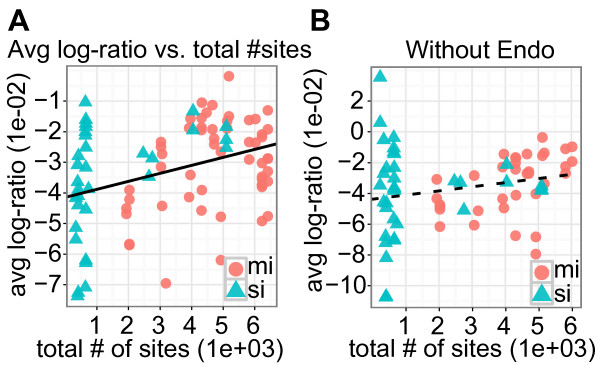
**Genes without target sites for endogenous miRNAs show less dilution effects than does the complete set of potential targets**. (**A**) The scatter plot shows the average log ratios for predicted miRNA and siRNA targets as measured by microarrays of 90 over-expression experiments (55 miRNAs and 35 siRNAs) as a function of the miRNAs' and siRNAs' total number of target sites. The line is based on a linear regression and indicates that there is a significant correlation between the total number of target sites and average log ratio (r = 0.37; *P *< 0.001). (**B**) The scatter plot shows the average log ratios for the subset of genes that have no predicted target sites for endogenous miRNAs as a function of the miRNAs' and siRNAs' total number of target sites (r = 0.22; *P *= 0.068). Only the 70 samples assayed in HeLa were included. In both plots, red circles represent miRNAs, and blue triangles represent siRNAs. miRNA, microRNA; siRNA, small interfering RNA.

A recent study has reported that when over-expressing miRNAs, a high overall expression level of predicted targets reduces the miRNA's average regulatory effect - the so-called target dilution effect [22]. Arvey and colleagues mainly used the total mRNA expression level to test the dilution effect, but they also suggested that other approaches were almost equally effective, such as the total number of target sites [22]. To test the dilution effect on the samples, we, therefore, used the number of target sites instead of total mRNA expression levels, as these values were very highly correlated (*r *= 0.973; Additional file 1, Figure S3) and also because mRNA expression data were unavailable for the HCT116 and DLD-1 cell-lines used in the Linsley experiment. Our results confirmed a significant correlation between the total number of target sites and the average log ratio of predicted miRNA targets (*r *= 0.369; *P*-value < 0.001; Figure 4A).

Targeting by endogenous miRNAs influences both the genes' expression levels before transfection (Figure 3D) and response to exogenous miRNAs after transfection (Figure 2). We therefore reasoned that part of the observed correlation could be related to interactions between endogenous miRNAs and the exogenous, ectopically expressed miRNA because some exogenous miRNA target genes also targeted by endogenous miRNAs were potentially up-regulated because of the competition effects. Such interactions could be further compounded by the microarrays' bias towards detecting differential expression among highly expressed genes (Figure 3E). Arvey and colleagues based their conclusions on microarray data and they also reported that for most transfected miRNAs or siRNAs (166 of 181 tested; *P*-value = 2e-33, sign test), highly expressed genes are more down-regulated than are lowly expressed genes [22]. To eliminate such interactions between the endogenous and exogenous miRNAs, we calculated the correlation between the total number of miRNA sites and the average log ratio of the genes that were predicted only to be targets for the exogenous miRNAs. The correlation was not significant (*r *= 0.22; *P*-value = 0.067; Figure 4B), indicating that when considering the average effects of exogenous, ectopically expressed miRNAs, endogenous miRNA regulation (competition [21]) is more important than overall target expression levels (dilution).

### Regression analysis: linear regression confirmed trends from individual feature analyses

To investigate further how the different features collectively contributed to log ratio changes of gene expression, we built a linear regression model with eight factors per mRNA target. These eight factors represented our previous target, sample, and platform level features (Table 3). To create the model, we first calculated the eight factors for all predicted miRNA or siRNA target genes and transformed the factors' value range to [0, 1], to make regression coefficient values easily comparable. Second, we negated all log-ratio values for the transfection experiments so that a positive coefficient meant that a high value for the factor contributed positively to gene down-regulation. Third, we built a linear regression model with the eight factors on the set of predicted miRNA and siRNA target genes from the ten experiments (R^2 ^= 0.040; R^2 ^= 0.040, when adjusted by the number of records and the number of terms).

**Table 3:** Nine factors for linear regression

Factor	Description	Values
ln3	Target's 3' UTR length	0~1
cs3	Target's overall 3' UTR conservation	0~1
exp	Target's mRNA expression level as measured by RNA-Seq.	0~1
#site_m	The number of miRNA or siRNA target sites in the target's 3' UTR	0~1
#endo_m	The number of target sites for endogenous miRNAs in the target's 3' UTR	0~1
#site_s	The total number of miRNA or siRNA target sites in all the potential target 3' UTRs	0~1
p_ma	Binary variable stating whether the target's fold change was measured by microarray or proteomics	1: Microarray
		0: Proteomics
e_oe	Binary variable stating whether the target's fold change was measured after miRNA transfection or inhibition	1: Transfection
		0: Inhibition
ts_score	mRNA level TargetScan scores. Only used in the last regression model.	0~1

All values are either in a range between 0 and 1 (0~1) or binary (0 or 1). miRNA, microRNA; mRNA, messenger RNA; siRNA, small interfering RNA; UTR, untranslated region.

**Figure 5:**
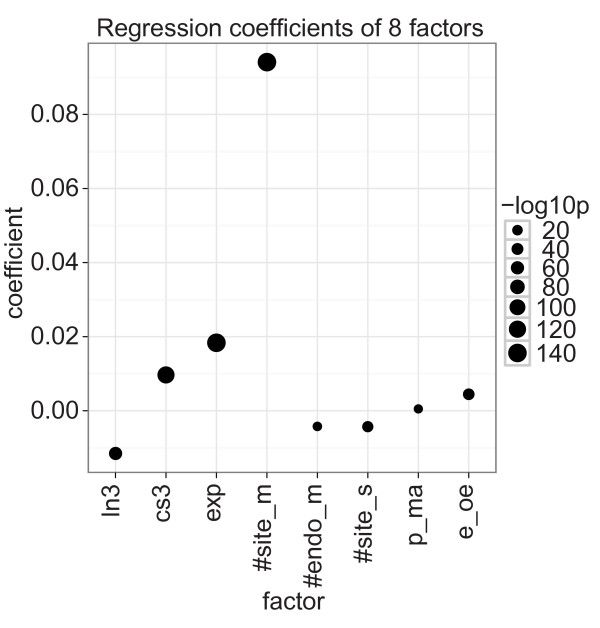
**Coefficients of a linear regression with eight factors**. The dot plot shows the coefficients of the liner model with formula: -log ratio = ln3 + cs3 + exp +#site_m + #endo_m + #site_s + p_ma + e_oe. The dot size shows -log_10 _of the coefficient's *P*-value. Positive coefficients associate with miRNA down-regulation. miRNA, microRNA.

The model showed that seven of the factors significantly contributed to log ratio changes, although to different extents (Figure 5; Additional file 1, Table S17). Consistent with published results that multiple miRNA target sites enhance miRNA down-regulation [27], the number of target sites (#site_m) was the strongest factor, and consistent with our non-parametric tests, mRNA expression (exp) had the second largest coefficient. Furthermore, 3' UTR length (ln3), the number of target sites for endogenous miRNAs (#endo_m), and the total number of target sites per sample (#site_s) had strong negative coefficients, whereas transfection compared with inhibition experiments (e_oe) had, as expected, a positive coefficient. Partly contrary to the non-parametric analyses, however, 3' UTR conservation (cs3) was consistently and strongly associated with target knock-down, but this result likely reflects the fact that highly conserved 3' UTRs were strongly associated with target knock-down in four of the ten experiments (Figure 1).

### Regression analysis: factor crossing in the linear model confirmed that several factors have combined effects on miRNA down-regulation

Based on our analyses of the individual target, sample, and experiment features, we expected that some factor pairs such as mRNA expression (exp) and proteomics compared with microarray experiments (p_ma), the number of target sites for the exogenous small RNA (#site_m) and endogenous miRNA (#endo_m), and 3' UTR conservation (cs3) and transfection compared with inhibition experiments (e_oe) had strong combined effects. To investigate this possibility, we extended our simple linear model to include all second-order interactions, such as ln3 * cs3, ln3 * exp, and so on. This extension slightly increased the R^2 ^of the model (R^2 ^= 0.047; adjusted R^2 ^= 0.046).

Although directly comparing coefficients was less straightforward than for the simpler model without factor interactions because of different range distributions for combined factors, there were still several factors that had significantly higher or lower coefficients than the others (Additional file 1, Figure S4 and Table S18). The number of target sites for the ectopically expressed miRNA or siRNA (#site_m) had the highest coefficient, whereas the interaction between #site_m and the number of target sites for endogenous miRNAs (#endo_m) had the most negative and most significant coefficient. Consistent with the importance of the number of target sites for regulation, most factor combinations that included #site_m were significant. These results confirm that the number of target sites (#site_m) is the most important factor to explain log ratio changes, but that several other interacting factors and especially competition with endogenous miRNAs (#endo_m) influence the resulting target knock-down.

Of the other factors, target expression (exp) was still among the most significant single factor. Moreover, consistent with our previous results, the coefficients for exp's interaction with the two experiment factors (p_ma and e_oe) were strong, significant, and positive. In contrast, exp by itself or interacting with 3' UTR length (ln3) had a significant negative coefficient. Consequently, our results suggest that when experiment-related features are factored out, miRNAs do more strongly affect lowly than highly expressed genes.

As for the remaining features, both ln3 and cs3 showed significant interactions with expected features. Especially cs3 showed strong and significant interactions with many factors (all except ln3, exp, and p_ma), which likely explains the conflicting results for 3' UTR conservation in the individual feature analyses.

### Regression analysis: target site-dependent features, as modeled by TargetScan scores, show strong interactions with site-independent features

So far, our regression models and analyses considered all stringent seed sites as equally important target sites (see Methods), but different features of individual target sites, such as the seed type, the site's AU context, the site's location in 3' UTR, and additional pairing between the miRNA 3' end and mRNA, do affect miRNA targeting [18]. We therefore extended our regression model to include TargetScan [18] scores (see Methods) to determine how the predicted effects of individual miRNA target sites affect and interact with the mRNA, sample, and platform level features in our model.

**Figure 6:**
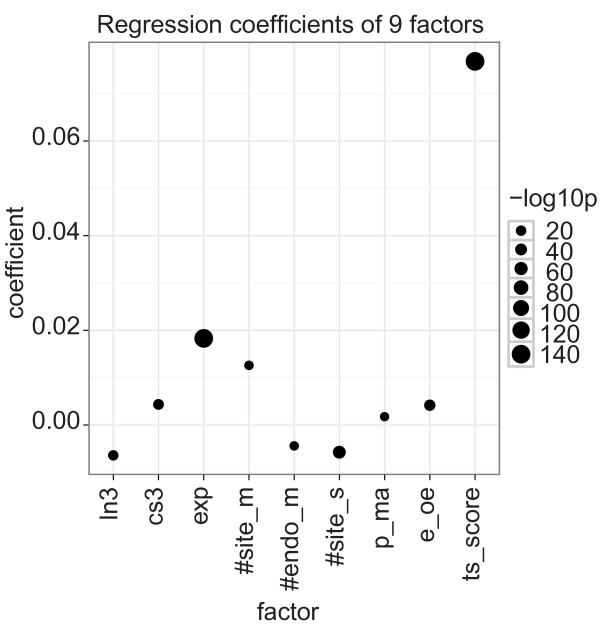
**Coefficients of a linear regression with nine factors**. The dot plot shows the coefficients of the liner model with formula: -log ratio = ln3 + cs3 + exp +#site_m + #endo_m + #site_s + p_ma + e_oe + ts_score. The dot size shows -log_10 _of the coefficient's p-value. Positive coefficients associate with miRNA down-regulation. miRNA, microRNA.

A simple regression model with only TargetScan scores had R^2 ^= 0.043, which was very similar to the previous models with eight factors (R^2 ^= 0.040 and R^2 ^= 0.046 for the simple and combined effects models, respectively). In contrast, a simple regression model that included all the nine factors showed increased R^2 ^(R^2 ^= 0.071; adjusted R^2 ^= 0.071). The most significant single factor in this model was TargetScan score (ts_score; Figure 6; Additional file 1, Table S19). Most factors showed very similar trends compared with the previous model with eight factors (Figure 5), except for the number of target sites for the ectopically expressed miRNA or siRNA (#site_m) which showed decreased importance. This decrease can be explained, however, by TargetScan modeling total mRNA regulation as the sum of the scores for individual target sites; TargetScan scores (ts_score) and the number of target sites for the ectopically expressed miRNA or siRNA (#site_m) are strongly correlated (*r *= 0.42; *P*-value < 2.2e-16).

Taking all second-order interactions into account further improved the model (R^2 ^= 0.093; adjusted R^2 ^= 0.092) and TargetScan score combined with most other factors (except #site_m) showed significant coefficients (Additional file 1, Figure S5 and Table S20). Together, these results show that although target site-dependent features, such as those modeled by TargetScan, are important for miRNA targeting, target mRNA, sample, and platform level features are also important for correctly interpreting miRNA high-throughput experiments. Specifically, in our analyses, target site-dependent and -independent features explain about the same amount of variation in target gene expression.

### CpG frequency, and developmental and housekeeping genes: overall gene expression is a major confounding factor when analyzing microarray data

**Figure 7:**
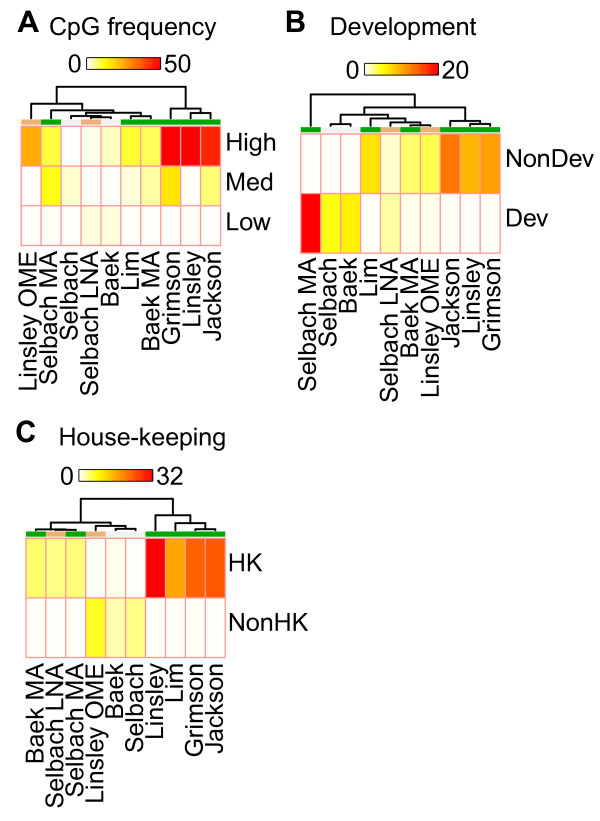
**CpG-rich genes, non-developmental genes, and housekeeping genes appear to be strong miRNA targets in microarray experiments**. We subdivided all RefSeq genes into sub-groups based on three different features: CpG frequency (CpG), and whether the genes were developmental (Dev) and housekeeping (HK) genes (see Methods). See Figure 1 for a description of the heat maps. miRNA, microRNA.

As studies reported that miRNAs preferably target the high-CpG (CpGH) genes [14] and developmentally regulated (Dev) genes [3] and also tend to avoid targeting housekeeping (HK) genes [2,8], we expected the CpGH, Dev, and non-housekeeping (Non-HK) genes to be strongly down-regulated in the experimental data. To test miRNA down-regulation of these features, we split them into smaller sub-groups and used predicted miRNA or siRNA target genes to test whether any sub-group was more down-regulated than the others (Figure 7). As expected, the CpGH genes were significantly affected (Figure 7A; Additional file 1, Table S21), but we observed the opposite to the expected for the Dev and Non-HK genes (Figure 7B, C; Additional file 1, Tables S22 and S23). Importantly, only the microarray data showed these unexpected differential effects. The proteomics data showed slightly stronger effects on the Dev and Non-HK genes, although only one of four comparisons was significant (Additional file 1, Tables S22 and S23). We, therefore, reasoned that the results could be related to the bias of the microarrays toward detecting differential expression among highly expressed genes.

Consistent with this hypothesis, non-parametric tests to find the characteristics of CpG, HK, and Dev showed that CpGH and HK genes had more highly expressed genes than the other sub-groups and also showed that Dev genes had more lowly expressed genes (Table 4). We, therefore, concluded that the overall expression level of the genes was the major factor behind the observed differences within the CpG, Dev, and HK groups.

**Table 4:** *P*-values of multiple Wilcoxon rank-sum tests on three miRNA target features for CpGH, House-keeping, and Developmental genes

Gene type	Feature	Greater		Less	
CpGH	3' UTR Length	3.85E-096	*	1	
	3' UTR Consv	2.13E-206	*	1	
	mRNA Exp	0	*	1	
Development	3' UTR Length	8.46E-017	*	1	
	3' UTR Consv	2.11E-051	*	1	
	mRNA Exp	0.989438416		0.010561646	*
Housekeeping	3' UTR Length	1		1.89E-020	*
	3' UTR Consv	0.469643425		0.530357925	
	mRNA Exp	1.57E-091	*	1	

Lower *P*-values in the Greater column indicate that the gene set of the Gene type overall has longer 3' UTR lengths, more conserved 3' UTRs, or stronger mRNA expression than has the rest of the human protein coding genes. Lower *P*-values in the Less column indicates the opposite characteristics; that is, shorter 3' UTR lengths, less conserved 3' UTRs, or lower mRNA expression. CpGH, high-CpG; Consv, conservation; Exp, expression; miRNA, microRNA; mRNA, messenger RNA; UTR, untranslated region.

## Conclusions

We analyzed the average regulatory effects that ectopically expressed miRNAs or siRNAs have on large gene sets and identified two strong factors. First, competition between endogenous miRNAs and the ectopically expressed RNAs have a strong impact on the targets' regulatory response. Genes with very long 3' UTRs, for example, are likely targeted by endogenous miRNAs and therefore are less affected by exogenous miRNAs than are genes with shorter 3' UTRs. Second, target gene expression is a strong confounding factor when analyzing microarray experiments. Target genes with strong expression levels were significantly down-regulated compared to other expressed genes only in the microarray experiments. One explanation is that some genes are highly expressed simply because they are less targeted by endogenous miRNAs, and, therefore, ectopically expressed miRNAs have stronger down-regulating effects on these genes. Consistent with this explanation, miRNA transfection experiments give stronger target expression changes than do miRNA inhibition experiments.

Another explanation is that microarrays, compared with high throughput proteomics, report significant expression data for many more lowly expressed genes. This technological difference means that microarrays can detect more differentially expressed genes than can high throughput proteomics. However, microarrays generally detect less differential expression for lowly expressed genes than for highly expressed genes. Consequently, microarrays will find a smaller fraction of the lowly expressed genes than of the highly expressed genes to be differentially expressed. The result is that when using microarray data to compare average down-regulation of miRNA-targets among housekeeping genes and developmentally regulated genes - genes that have high and low average expression levels, respectively - the average down-regulation is stronger for housekeeping genes than for developmental genes. This difference is contrary to the general consensus within the field [7] and to our analyses of high-throughput proteomics data which indicate that developmentally regulated genes are more likely miRNA targets than are housekeeping genes. Supporting this, our regression analysis shows that when such experiment-related confounding factors have been taken into account, miRNAs more effectively knock down lowly expressed than highly expressed genes.

These results suggest that it is important to consider multiple factors when it comes to assessing miRNA targeting effects. One example of this relates to the so-called target dilution effect. A previous analysis has reported that the total expression level of target candidates for ectopically expressed miRNAs affects the targets' average knockdown such that high total expression gives low average knockdown [22]. Our analyses, however, show that some of this dilution effect can be explained by interactions with endogenous miRNAs. Consequently, it is very important to consider what genes are already targeted by endogenous miRNAs when designing and interpreting high throughput miRNA or siRNA experiments.

In summary, our results can explain the results from several recent studies that have analyzed features that are important for miRNA regulation and found that the importance of 3' UTR length, conservation, and target gene expression depend on the technology used to measure miRNA targeting. Our results urge special caution when using microarray data to compare average regulatory effects between groups of genes that have different average gene expression levels, such as high and low CpG genes and housekeeping and developmentally regulated genes.

## Methods

### Data retrieval

#### miRNA annotation and miRNA seed types

We downloaded the annotations of human miRNAs, mature miRNAs, and miRNA families from miRBase (release 12.0) [29].

#### Microarray and proteomics datasets

We downloaded four microarray datasets, the Jackson [24], Lim [17], Grimson [18], and Linsley [30] from the Gene Expression Omnibus (GEO) database [GEO:GSE5814, GEO:GSE2075, GEO:GSE8501, GEO:GSE6838] [31], and two proteomics datasets, Selbach [19] and Baek [20], from the original publications along with the corresponding microarray datasets. In total, we used six microarray and two proteomics datasets. Samples from both microarray and proteomics used in this study are listed in Additional file 1, Table S2.

#### 3' UTR sequence and conservation

We downloaded the RefSeq transcripts (hg18), human chromosome sequences, and multiz 17-way [32] for conserved sequences from the University of California, Santa Cruz (UCSC) Genome Browser [33]. The positional data used to generate nucleotide sequences were the exon positions from RefSeq for the 3' UTR region. We selected the longest 3' UTRs when a RefSeq entry had multiple transcripts. In addition to the human sequences, we generated 3' UTR sequences conserved in human, mouse, and rat (HMR) from multiz 17-way.

#### mRNA expression in HeLa

We used replicate 1 of the ENCODE Caltech RNA-Seq data [25] from USCS for the mRNA expression in HeLa.

#### Housekeeping and developmental genes

We obtained housekeeping genes from a list generated by a Naive Bayes classifier [34], and the developmental genes from the Gene Ontology [GO:0032502] [35].

#### Endogenous miRNAs in HeLa

We used the Mammalian microRNA Expression Atlas [36] to define the endogenous miRNAs in HeLa. For endogenous miRNAs, we selected the top ten highly expressed miRNA families and used the miRNAs that belong to these miRNA families. We used three stringent seed types - 8mer, 7mer-A1, and 7mer-m8 - to search the candidate sites of the endogenous miRNAs.

#### PAR-CLIP

We obtained PAR-CLIP high-throughput sequencing data of all four AGO proteins from the GEO database [GEO:GSE21918] [16]. We used positional information downloaded with the sequence reads and mapped these positions on 3' UTR regions of all RefSeq genes. We considered 3' UTRs that had at least one site with the number of mapped reads greater than or equal to five to have an AGO binding site.

### Data preparation

#### Data preparation for microarray and proteomics data

We used pre-processed data either from GEO or from the original publications. All log-ratio values that were pre-computed in log_2 _were transformed to log_10_. Log-ratio values of two inhibition experiments, Selbach LNA and Linsley OME, were negated because genes with positive log-ratio values were potential miRNA targets for these experiments.

#### Predicted miRNA or siRNA targets

We separated the genes of each high throughput experiment by target prediction into 'Target' and 'Non-target' genes. The target prediction method we used was a simple stringent seed search on the 3' UTRs and a gene was defined as a 'Target' when the gene contained a seed site for the miRNA or siRNA used in the experiment. We used the previously described three stringent seed types, 8mer, 7mer-A1, and 7mer-m8, to define miRNA targets [7].

#### 3' UTR sequence length

For the 3' UTR sequence length, we made five sub-groups, Very Long (> 4,000 nts), Long (1,373 to approximately 4,000 nts), Medium Long (630 to approximately 1,372 nts), Medium Short (248 to approximately 629 nts), and Short (0 to approximately 247 nts). The first group, Very Long, was decided from our previous study [13] because it showed that the genes with 3' UTR length longer than 4,000 nucleotides were less targeted by miRNAs. We sorted the rest of the sequences by 3' UTR length and divided them into four equally sized sub-groups.

#### 3' UTR sequence conservation

For the 3' UTR sequence conservation, we calculated the conservation scores for each sequence by counting the number of conserved nucleotides in the HMR sequences and then divided the resulting number by the length of the sequence. We sorted the sequences with non-zero scores and divided them into three equal sub-groups; High (> 0.238), Medium (0.054 to approximately 0.238), and Low (0 to approximately 0.054). The sequences with zero scores were categorized as NoConsv.

#### mRNA expression level

We used the average number of tags from Caltech RNA-Seq as a measure (score) of mRNA expression levels. We sorted the mRNAs with non-zero expression scores by score and then divided them into five equally sized sub-groups; Very High (> 0.2007), High (0.0795 to approximately 0.2007), Medium (0.0344 to approximately 0.0795), Low (0.008 to approximately 0.0344), and Very Low (0 to approximately 0.008). The mRNAs with zero scores were categorized as NoExp.

#### CpG frequency in promoters

We defined the promoter regions as 1,000 nucleotides upstream from the transcription start site. We used a moving window approach (500 nt window moving 5 nt at a time) to compute the CpG frequency and classified the CpG frequency as 'high' when at least one 500-nucleotide-window contained > 55% GC content and > 75% CpG content, 'low' when none of the windows contained > 48% CpG content, and 'medium' for the rest [37].

#### Housekeeping and developmental genes

We mapped housekeeping and developmental genes to RefSeq genes based on gene IDs. Non-housekeeping and non-developmental genes were the rest of the RefSeq genes that were not mapped.

#### Endogenous miRNA targets

We split 'Target' and 'Non-Target' genes into 'With endogenous' and 'Without endogenous' to make the T +Endo, T -Endo, NT +Endo, and NT -Endo gene groups, where 'T' and 'NT' represent 'Target' and 'Non-Target', whereas '+Endo' and '-Endo' represent 'With endogenous' and 'Without endogenous'. We defined a gene as 'With endogenous' when the gene was a predicted targeted for one or more of the top ten most highly expressed endogenous miRNA families. The same approach as for predicting miRNA and siRNA targets was used to predict target genes for endogenous miRNAs.

#### Total mRNA expression levels and total number of target sites for the dilution effects

We calculated both the total mRNA expression and the number of target transcripts as previously described [22]. Specifically, the total mRNA expression was a sample level sum of the average number of tags from Caltech RNA-Seq for predicted miRNA or siRNA target genes. The total number of target sites was a sample level sum of the number of target sites in predicted miRNA or siRNA target genes.

#### mRNA level TargetScan scores

The stand alone version of TargetScan was downloaded from the TargetScan website http://www.targetscan.org. We then ran TargetScan on the 3' UTR sequences of the genes from the ten miRNA high-throughput experiments with corresponding miRNA or siRNA sequences. The scores of target sites were aggregated by miRNA:mRNA pairs, and the aggregated scores were negated. Therefore, a high mRNA-level TargetScan score indicates that the mRNA is a strong candidate for miRNA down-regulation.

### Statistical analysis

#### Non-parametric tests

We used the log-ratio values from 10 different experiments (Additional file 1, Table S1) that contain 140 different samples (Additional file 1, Table S2) to measure the contributions of different groups to miRNA targeting efficacy. To test the significance level between multiple groups, we performed both one-sided Wilcoxon rank-sum and one-sided Kolmogorov-Smirnov non-parametric multiple comparison tests on the log-ratio values.

#### Sample level scores

We performed non-parametric tests on 140 samples and counted the number of samples that had a significant *P*-value (< 0.05) to calculate the proportion per experiment as Sample level score.

#### PAR-CLIP analysis

We merged the five sub-groups of 3' UTR lengths into the two bigger groups long (Very Long and Long) and short (Med Long, Med Short, and Short) for all RefSeq genes and identified potential miRNA target genes bound by the four AGOs. The numbers of bound genes in the long and short groups were compared between each AGO and all RefSeq genes by Fisher's exact test.

#### Log_2 _enrichment of down-regulated genes

The enrichment was calculated as the average log ratio values of down-regulated genes (*P*-value < 0.01 and log ratio < -0.01 for microarray, and log ratio < -0.01 for proteomics) divided by the average log-1ratio values of all genes.

#### Linear regression

All factors were normalized to the [0, 1] value range before building linear regression models. The normalization was linear; that is, (feature value - min)/(max - min), where min and max values were defined for each factor as ln (min: -2.0, max: 2.0), ln3 (min: 0, max: 1000), cs3 (min:0, max: 1), exp (min:6.1e-5, max: 64.0), #site_m (min: 0, max: 20), #endo_m (min: 0, max: 30), #endo_s (min: 0, max: 6000), and ts_score (min: 0.0, max: 2.0).

## Abbreviations

CLIP: cross-linked immunoprecipitation; CpGH: high-CpG; Dev: developmentally regulated; GEO: Gene Expression Omnibus; HK: housekeeping; LNA: locked nucleic acid; miRNA: microRNA; mRNA: messenger RNA; ncRNA: non-coding RNA; nts: nucleotides; OME: 2'-O-methyl; PAR-CLIP: Photoactivatable-Ribonucleoside-Enhanced Crosslinking and Immunoprecipitation; RIP: RNA immunoprecipitation; siRNA: small interfering RNA; UCSC: University of California: Santa Cruz; UTR: untranslated region.

## Competing interests

The authors declare that they have no competing interests.

## Authors' contributions

All authors contributed to the underlying ideas of the method and the analysis. TS implemented the initial model of statistical analyses. All authors contributed to the statistical analysis. The initial manuscript draft was written by TS, and refined by PS. All authors read and approved the final manuscript.

## Supplementary Material

Additional file 1 **Supplementary information**. Additional file 1 contains Supplementary Tables S1-S23 and Supplementary Figures S1-S5. Click here for file
