# Supplementary material for: Target gene expression levels and competition between transfected and endogenous microRNAs are strong confounding factors in microRNA high-throughput experiments
Source: Silence. 2012 Feb 10;3:3. doi: 10.1186/1758-907X-3-3 (PMC3293725; doi:10.1186/1758-907X-3-3)
Supplement: Additional file 1 — Supplementary information. Additional file 1 contains Supplementary Tables S1-S23 and Supplementary Figures S1-S5. [file 1758-907X-3-3-S1.PDF]

Supplementary information

Target gene expression levels and competition between transfected and endogenous microRNAs are strong confounding factors in microRNA high-throughput experiments

Takaya Saito and Pål Sætrom

Contents

Supplementary Tables **3**
Table S1. Summary of miRNA high-throughput experiments 3
Table S2. Samples and miRNAs of miRNA high-throughput experiments 4
Table S3. P-values of multiple Wilcoxon rank-sum tests on 5 subgroups of 3' UTR length 7
Table S4. P-values of multiple Wilcoxon rank-sum tests on 4 subgroups of 3' UTR conservation 8
Table S5. P-values of multiple Wilcoxon rank-sum tests on 6 subgroups of mRNA expression 9
Table S6. P-values of multiple Kolmogorov–Smirnov tests on 5 subgroups of 3' UTR length 10
Table S7. P-values of multiple Kolmogorov–Smirnov tests on 4 subgroups of 3' UTR conservation 11
Table S8. P-values of multiple Kolmogorov–Smirnov tests on 6 subgroups of mRNA expression 12
Table S9. Sample level scores of Wilcoxon rank-sum tests on 3' UTR length 13
Table S10. Sample level scores of Wilcoxon rank-sum tests on 3' UTR conservation 14
Table S11. Sample level scores of Wilcoxon rank-sum tests on mRNA expression. 15
Table S12. The number of genes for four gene groups, T -Endo, T +Endo, NT -Endo, and NT -Endo, per experiment 16
Table S13. P-values of multiple Wilcoxon rank-sum tests between T -Endo and T +Endo on 5 subgroups of 3' UTR length 17
Table S14. P-values of multiple Wilcoxon rank-sum tests between T -Endo and T +Endo on 5 subgroups of 3' UTR conservation 18
Table S15. P-values of multiple Wilcoxon rank-sum tests between T -Endo and T +Endo on 5 subgroups of mRNA expression 19
Table S16. Comparisons of Wilcoxon rank-sum tests for T -Endo against T +Endo between experimental and individual sample levels 20
Table S17. Coefficients and p-values of linear regression with eight factors 21
Table S18. Coefficients and p-values of linear regression with eight factors and factor crossing 22
Table S19. Coefficients and p-values of linear regression with nine factors 23
Table S20. Coefficients and p-values of linear regression with nine factors and factor crossing 24
Table S21. P-values of multiple Wilcoxon rank-sum tests on 3 subgroups of CpG frequency 25
Table S22 P-values of multiple Wilcoxon rank-sum tests on 2 subgroups of developmental genes 26
Table S23. P-values of multiple Wilcoxon rank-sum tests on 2 subgroups of housekeeping genes 27

Supplementary Figures **28**
Figure S1. mRNA expression levels from 6 microarray and 2 proteomics experiments. 28
Figure S2. Log2 enrichment of down-regulated genes compared with all genes mRNA. 29
Figure S3. Scatter plot of the total tag counts versus total number of miRNA target sites. 30
Figure S4. Coefficients of a linear regression with eight factors and factor crossing 31
Figure S5. Coefficients of a linear regression with nine factors and factor crossing 32

Supplementary Tables

Table S1. Summary of miRNA high-throughput experiments

* Type: types of experiments as Micrroarray overexpression (M), Proteomics overexpression (P), Microarray inhibition (M_OME), and Proteomics inhibition (P_LNA).

Experiment	# of unique miRNAs	# of samples	Type *
Baek MA	3	3	M
Baek	3	3	P
Lim	7	14	M
Jackson	26	66	M
Linsley	9	23	M
Linsley OME	2	2	M_OME
Grimson	9	18	M
Selbach LNA	1	1	P_LNA
Selbach MA	5	5	M
Selbach	5	5	P

Table S2. Samples and miRNAs of miRNA high-throughput experiments

* Sample ID: GEO Sample IDs for microarray experiments. Sample IDs are equivalent with Internal miRNA IDs for Baek MA, Baek, Selbach LNA, Selbach MA, and Selbach.

Experiment	Sample ID *	Internal miRNA ID
Baek MA	baek_m_mir_1	baek_m_mir_1
Baek MA	baek_m_mir_124	baek_m_mir_124
Baek MA	baek_m_mir_181	baek_m_mir_181
Baek	baek_p_mir_1	baek_p_mir_1
Baek	baek_p_mir_124	baek_p_mir_124
Baek	baek_p_mir_181	baek_p_mir_181
Lim	GSM37602	mirna-lim2005-chimiR-1/124
Lim	GSM37603	mirna-lim2005-chimiR-1/124
Lim	GSM37604	mirna-lim2005-chimiR-124/1
Lim	GSM37605	mirna-lim2005-chimiR-124/1
Lim	GSM37598	mirna-lim2005-miR-1
Lim	GSM37599	mirna-lim2005-miR-1
Lim	GSM37600	mirna-lim2005-miR-124
Lim	GSM37601	mirna-lim2005-miR-124
Lim	GSM37606	mirna-lim2005-miR-124mut5-6
Lim	GSM37607	mirna-lim2005-miR-124mut5-6
Lim	GSM37608	mirna-lim2005-miR-124mut9-10
Lim	GSM37609	mirna-lim2005-miR-124mut9-10
Lim	GSM37610	mirna-lim2005-miR-373
Lim	GSM37611	mirna-lim2005-miR-373
Jackson	GSM133702	sirna-jackson2006wso-1
Jackson	GSM134468	sirna-jackson2006wso-1
Jackson	GSM133701	sirna-jackson2006wso-10
Jackson	GSM134485	sirna-jackson2006wso-10
Jackson	GSM134508	sirna-jackson2006wso-10
Jackson	GSM133700	sirna-jackson2006wso-11
Jackson	GSM134484	sirna-jackson2006wso-11
Jackson	GSM134507	sirna-jackson2006wso-11
Jackson	GSM134483	sirna-jackson2006wso-12
Jackson	GSM134506	sirna-jackson2006wso-12
Jackson	GSM133699	sirna-jackson2006wso-13
Jackson	GSM134482	sirna-jackson2006wso-13
Jackson	GSM134505	sirna-jackson2006wso-13
Jackson	GSM133698	sirna-jackson2006wso-14
Jackson	GSM134481	sirna-jackson2006wso-14
Jackson	GSM134504	sirna-jackson2006wso-14
Jackson	GSM133697	sirna-jackson2006wso-15
Jackson	GSM134480	sirna-jackson2006wso-15
Jackson	GSM134503	sirna-jackson2006wso-15
Jackson	GSM133696	sirna-jackson2006wso-16
Jackson	GSM134479	sirna-jackson2006wso-16
Jackson	GSM134502	sirna-jackson2006wso-16
Jackson	GSM133695	sirna-jackson2006wso-17
Jackson	GSM134478	sirna-jackson2006wso-17
Jackson	GSM134501	sirna-jackson2006wso-17
Jackson	GSM133686	sirna-jackson2006wso-18
Jackson	GSM134477	sirna-jackson2006wso-18
Jackson	GSM134500	sirna-jackson2006wso-18
Jackson	GSM133694	sirna-jackson2006wso-19
Jackson	GSM134476	sirna-jackson2006wso-19
Jackson	GSM134499	sirna-jackson2006wso-19
Jackson	GSM133685	sirna-jackson2006wso-20

Jackson	GSM134475	sirna-jackson2006wso-20
Jackson	GSM134498	sirna-jackson2006wso-20
Jackson	GSM133693	sirna-jackson2006wso-21
Jackson	GSM134474	sirna-jackson2006wso-21
Jackson	GSM134497	sirna-jackson2006wso-21
Jackson	GSM133692	sirna-jackson2006wso-22
Jackson	GSM134473	sirna-jackson2006wso-22
Jackson	GSM134496	sirna-jackson2006wso-22
Jackson	GSM133691	sirna-jackson2006wso-23
Jackson	GSM134472	sirna-jackson2006wso-23
Jackson	GSM134495	sirna-jackson2006wso-23
Jackson	GSM133690	sirna-jackson2006wso-24
Jackson	GSM134471	sirna-jackson2006wso-24
Jackson	GSM134494	sirna-jackson2006wso-24
Jackson	GSM133687	sirna-jackson2006wso-25
Jackson	GSM134470	sirna-jackson2006wso-25
Jackson	GSM134493	sirna-jackson2006wso-25
Jackson	GSM133684	sirna-jackson2006wso-26
Jackson	GSM134469	sirna-jackson2006wso-26
Jackson	GSM134492	sirna-jackson2006wso-26
Jackson	GSM134467	sirna-jackson2006wso-27
Jackson	GSM134466	sirna-jackson2006wso-28
Jackson	GSM134488	sirna-jackson2006wso-30
Jackson	GSM134491	sirna-jackson2006wso-31
Jackson	GSM134511	sirna-jackson2006wso-4
Jackson	GSM134512	sirna-jackson2006wso-5
Jackson	GSM133688	sirna-jackson2006wso-7
Jackson	GSM134487	sirna-jackson2006wso-7
Jackson	GSM134489	sirna-jackson2006wso-7
Jackson	GSM134510	sirna-jackson2006wso-7
Jackson	GSM133689	sirna-jackson2006wso-8
Jackson	GSM134486	sirna-jackson2006wso-8
Jackson	GSM134490	sirna-jackson2006wso-8
Jackson	GSM134509	sirna-jackson2006wso-8
Linsley	GSM156557	mirna-linsley2007-let-7c
Linsley	GSM156558	mirna-linsley2007-let-7c
Linsley	GSM156544	mirna-linsley2007-miR-103
Linsley	GSM156580	mirna-linsley2007-miR-103
Linsley	GSM156523	mirna-linsley2007-miR-15a
Linsley	GSM156545	mirna-linsley2007-miR-15a
Linsley	GSM156549	mirna-linsley2007-miR-15a
Linsley	GSM156577	mirna-linsley2007-miR-15a
Linsley	GSM156522	mirna-linsley2007-miR-16
Linsley	GSM156546	mirna-linsley2007-miR-16
Linsley	GSM156550	mirna-linsley2007-miR-16
Linsley	GSM156579	mirna-linsley2007-miR-16
Linsley	GSM156553	mirna-linsley2007-miR-17-5p
Linsley	GSM156555	mirna-linsley2007-miR-17-5p
Linsley	GSM156547	mirna-linsley2007-miR-192
Linsley	GSM156551	mirna-linsley2007-miR-192
Linsley	GSM156554	mirna-linsley2007-miR-20
Linsley	GSM156556	mirna-linsley2007-miR-20
Linsley	GSM156548	mirna-linsley2007-miR-215
Linsley	GSM156552	mirna-linsley2007-miR-215
Linsley	GSM156524	mirna-linsley2007_miR_106b
Linsley	GSM156543	mirna-linsley2007_miR_106b
Linsley	GSM156576	mirna-linsley2007_miR_106b
Linsley OME	GSM155603	mirna-linsley2007OME-miR-16
Linsley OME	GSM155605	mirna-linsley2007OME_miR_106b
Grimson	GSM210900	mirna-grimson2007-miR-122a

Grimson	GSM210901	mirna-grimson2007-miR-122a
Grimson	GSM210902	mirna-grimson2007-miR-128a
Grimson	GSM210903	mirna-grimson2007-miR-128a
Grimson	GSM210904	mirna-grimson2007-miR-132
Grimson	GSM210905	mirna-grimson2007-miR-132
Grimson	GSM210906	mirna-grimson2007-miR-133a
Grimson	GSM210907	mirna-grimson2007-miR-133a
Grimson	GSM210908	mirna-grimson2007-miR-142-3p
Grimson	GSM210909	mirna-grimson2007-miR-142-3p
Grimson	GSM210910	mirna-grimson2007-miR-148a
Grimson	GSM210911	mirna-grimson2007-miR-148a
Grimson	GSM210912	mirna-grimson2007-miR-181a
Grimson	GSM210913	mirna-grimson2007-miR-181a
Grimson	GSM210896	mirna-grimson2007-miR-7
Grimson	GSM210897	mirna-grimson2007-miR-7
Grimson	GSM210898	mirna-grimson2007-miR-9
Grimson	GSM210899	mirna-grimson2007-miR-9
Selbach LNA	psilac_p_let_7	psilac_p_let_7
Selbach MA	psilac_m_let_7_oe	psilac_m_let_7_oe
Selbach MA	psilac_m_mir1	psilac_m_mir1
Selbach MA	psilac_m_mir155	psilac_m_mir155
Selbach MA	psilac_m_mir16	psilac_m_mir16
Selbach MA	psilac_m_mir30	psilac_m_mir30
Selbach	psilac_p_let_7_oe	psilac_p_let_7_oe
Selbach	psilac_p_mir1	psilac_p_mir1
Selbach	psilac_p_mir155	psilac_p_mir155
Selbach	psilac_p_mir16	psilac_p_mir16
Selbach	psilac_p_mir30	psilac_p_mir30

Table S3. P-values of multiple Wilcoxon rank-sum tests on 5 subgroups of 3' UTR length

Lower p-values indicate that a set of genes in one sub-group is more down-regulated than a set of genes in the rest of subgroups. "*" and "**" are added when the p-value is less than 0.05 and Sidak adjusted (n=50) 0.001, respectively.

Experiment	Very Long	Long		Med Long		Med Short		Short	
Grimson	1	0.0001097	**	1.49E-06	**	0.9987		0.877	
Linsley	1	1		1.34E-14	**	0.0001561	**	1.47E-08	**
Lim	1	1		2.53E-08	**	4.02E-17	**	0.001384	*
Jackson	1	0.9861		5.66E-07	**	2.98E-09	**	0.02786	*
Baek	0.6108	0.4996		0.6649		0.1129		0.7686	
Selbach	0.9963	0.9267		0.1218		0.005917	*	0.0206	*
Baek MA	1	0.9988		0.0001289	**	1.43E-11	**	0.01611	*
Selbach MA	1	0.3123		0.002443	*	0.004716	*	0.3854	
Selbach LNA	0.4236	0.2408		0.9639		0.1824		0.4064	
Linsley OME	0.998	0.7781		0.004476	*	0.2858		0.4708	

Table S4. P-values of multiple Wilcoxon rank-sum tests on 4 subgroups of 3' UTR conservation

Lower p-values indicate that a set of genes in one sub-group is more down-regulated than a set of genes in the rest of subgroups. "*" and "**" are added when the p-value is less than 0.05 and Sidak adjusted (n=40) 0.0013, respectively.

Experiment	High		Medium		Low		NoConsv	
Grimson	3.18E-91	**	1.73E-05	**	1		1	
Linsley	1.54E-140	**	9.71E-09	**	1		1	
Lim	0.9605		1		0.7262		1.68E-12	**
Jackson	2.00E-14	**	0.442		0.9999		0.9971	
Baek	0.4501		0.06762		0.9964		0.1565	
Selbach	0.04254	*	0.7226		0.5001		0.8695	
Baek MA	0.5789		0.9598		0.8468		0.003194	*
Selbach MA	6.11E-11	**	0.003409	*	1		0.9809	
Selbach LNA	0.07027		0.9596		0.02426	*	0.9181	
Linsley OME	0.9973		0.09355		0.2657		0.2918	

Table S5. P-values of multiple Wilcoxon rank-sum tests on 6 subgroups of mRNA expression

Lower p-values indicate that a set of genes in one sub-group is more down-regulated than a set of genes in the rest of subgroups. "*" and "**" are added when the p-value is less than 0.05 and Sidak adjusted (n=60) 0.00086, respectively.

† The expression levels were measured for the HeLa cells, but some other cell line was used in the Linsley experiment. It is included only for comparison purpose.

Experiment	Very High		High		Medium		Low		Very Low	NoExp
Grimson	2.42E-90	**	8.74E-62	**	8.63E-20	**	0.007846	*	1	1
Linsley†	4.98E-143	**	3.67E-91	**	5.32E-44	**	0.9826		1	1
Lim	1.51E-26	**	0.003186	*	0.2223		0.9964		1	1
Jackson	1.83E-50	**	6.25E-12	**	0.06033		0.001487	*	1	1
Baek	0.1497		0.1157		0.9956		0.1983		0.9733	0.534
Selbach	0.4526		0.8428		0.1576		0.03415	*	0.9953	0.9641
Baek MA	4.62E-23	**	0.00139	*	7.83E-06	**	0.3092		0.9998	1
Selbach MA	2.94E-27	**	1.49E-07	**	0.0004135	**	0.9689		1	1
Selbach LNA	0.9988		0.3202		0.1359		0.01038	*	0.2094	0.615
Linsley OME	0.06633		1.30E-11	**	6.57E-06	**	2.07E-09	**	1	1

Table S6. P-values of multiple Kolmogorov–Smirnov tests on 5 subgroups of 3' UTR length

Lower p-values indicate that a set of genes in one sub-group is more down-regulated than a set of genes in the rest of subgroups. "*" and "**" are added when the p-value is less than 0.05 and Sidak adjusted (n=50) 0.001, respectively.

Experiment	Very Long	Long		Med Long		Med Short		Short	
Grimson	0.9916	1.44E-06	**	0.0001501	**	0.3243		0.04871	*
Linsley	0.9712	0.9938		1.19E-15	**	0.0001545	**	6.07E-06	**
Lim	0.971	0.9478		4.99E-06	**	1.25E-14	**	0.0001177	**
Jackson	0.9979	0.848		1.12E-05	**	1.99E-06	**	0.001449	*
Baek	0.4815	0.683		0.8718		0.2318		0.488	
Selbach	0.9702	0.6291		0.2414		0.01213	*	0.06909	
Baek MA	0.9893	0.9797		0.0002018	**	4.00E-08	**	0.02661	*
Selbach MA	0.9962	0.35		0.0002901	**	0.0005103	**	0.1217	
Selbach LNA	0.7271	0.2991		0.9337		0.1318		0.4176	
Linsley OME	0.9239	0.4324		0.01998	*	0.1771		0.3337	

Table S7. P-values of multiple Kolmogorov–Smirnov tests on 4 subgroups of 3' UTR conservation

Lower p-values indicate that a set of genes in one sub-group is more down-regulated than a set of genes in the rest of subgroups. "*" and "**" are added when the p-value is less than 0.05 and Sidak adjusted (n=40) 0.0013, respectively.

Experiment	High		Medium		Low	NoConsv	
Grimson	5.73E-92	**	8.36E-07	**	0.9862	0.9883	
Linsley	4.24E-128	**	2.31E-13	**	0.9771	0.9449	
Lim	0.01429	*	0.9909		0.5264	9.28E-18	**
Jackson	8.90E-21	**	0.01791	*	0.5197	0.1588	
Baek	0.3584		0.04377	*	0.9078	0.3234	
Selbach	0.07206		0.9122		0.6764	0.9017	
Baek MA	0.503		0.9653		0.8533	0.002383	*
Selbach MA	1.01E-09	**	0.001672	*	1	0.479	
Selbach LNA	0.02364	*	0.9725		0.08733	0.8624	
Linsley OME	0.9865		0.05635		0.358	0.05104	

Table S8. P-values of multiple Kolmogorov–Smirnov tests on 6 subgroups of mRNA expression

Lower p-values indicate that a set of genes in one sub-group is more down-regulated than a set of genes in the rest of subgroups. "*" and "**" are added when the p-value is less than 0.05 and Sidak adjusted (n=60) 0.00086, respectively.

† The expression levels were measured for the HeLa cells, but some other cell line was used in the Linsley experiment. It is included only for comparison purpose.

Experiment	Very High		High		Medium		Low		Very Low	NoExp	
Grimson	4.40E-88	**	1.64E-57	**	1.73E-20	**	3.10E-05	**	0.9806	0.8833	
Linsley†	2.83E-155	**	1.57E-115	**	1.00E-50	**	0.345		0.997	0.1561	
Lim	2.53E-19	**	0.002125	*	0.001156	*	0.9981		0.8145	0.9976	
Jackson	3.03E-50	**	1.33E-17	**	2.40E-05	**	0.0002529	**	0.9815	0.1539	
Baek	0.14		0.1617		0.985		0.2345		0.7886	0.5328	
Selbach	0.06506		0.8938		0.2683		0.0112	*	1	0.7825	
Baek MA	5.61E-19	**	0.001805	*	2.06E-05	**	0.06406		0.9993	1	
Selbach MA	2.26E-29	**	6.36E-12	**	1.94E-09	**	0.09846		0.8288	0.001052	*
Selbach LNA	0.9962		0.5803		0.08177		0.02633	*	0.387	0.6022	
Linsley OME	0.007603	*	7.60E-13	**	1.11E-08	**	2.67E-09	**	0.6525	0.1083	

Table S9. Sample level scores of Wilcoxon rank-sum tests on 3' UTR length

We performed the same Wilcoxon rank-sum tests as in Table S3 on samples instead of experiments. We then counted the number of samples that had a significant p-value (<0.05) to calculate the proportion per experiment as Sample level score. "*" and "**" are added when the score is between 0 and 0.5, and >0.5, respectively.

Experiment	Very Long		Long		Med Long		Med Short		Short	
Grimson	0.06	*	0.39	*	0.39	*	0.17	*	0.06	*
Linsley	0		0.17	*	0.57	**	0.39	*	0.35	*
Lim	0		0		0.63	**	0.63	**	0.38	*
Jackson	0		0		0.21	*	0.39	*	0.12	*
Baek	0		0		0		0		0	
Selbach	0		0		0		0.2	*	0.2	*
Baek MA	0		0		0.33	*	1	**	0.33	*
Selbach MA	0		0.2	*	0.6	**	0.6	**	0.2	*
Selbach LNA	0		0		0		0		0	
Linsley OME	0		0		0.5	*	0		0	

Table S10. Sample level scores of Wilcoxon rank-sum tests on 3' UTR conservation

We performed the same Wilcoxon rank-sum tests as in Table S4 on samples instead of experiments. We then counted the number of samples that had a significant p-value (<0.05) to calculate the proportion per experiment as Sample level score. "*" and "**" are added when the score is between 0 and 0.5, and >0.5, respectively.

Experiment	High		Medium		Low		NoConsv	
Grimson	0.94	**	0.22	*	0		0	
Linsley	0.83	**	0.3	*	0		0.09	*
Lim	0.25	*	0		0		0.5	*
Jackson	0.42	*	0		0.06	*	0.03	*
Baek	0		0.33	*	0		0	
Selbach	0		0		0		0	
Baek MA	0		0		0		0.33	*
Selbach MA	0.8	**	0.6	**	0		0.2	*
Selbach LNA	0		0		1	**	0	
Linsley OME	0		0		0.5	*	0	

Table S11. Sample level scores of Wilcoxon rank-sum tests on mRNA expression.

We performed the same Wilcoxon rank-sum tests as in Table S5 on samples instead of experiments. We then counted the number of samples that had a significant p-value (<0.05) to calculate the proportion per experiment as Sample level score. "*" and "**" are added when the score is between 0 and 0.5, and >0.5, respectively.

† The expression levels were measured for the HeLa cells, but some other cell line was used in the Linsley experiment. It is included only for comparison purpose.

Experiment	Very High	High	Medium	Low	Very Low	NoExp
Grimson	0.83 **	0.89 **	0.5 *	0.33 *	0	0
Linsley†	1 **	1 **	0.91 **	0.09 *	0	0
Lim	0.88 **	0.13 *	0.25 *	0	0	0
Jackson	0.67 **	0.48 *	0.06 *	0.18 *	0	0
Baek	0	0	0	0	0	0
Selbach	0.2 *	0	0.2 *	0.2 *	0	0
Baek MA	1 **	0.67 **	0.67 **	0.33 *	0	0
Selbach MA	0.6 **	0.8 **	0.6 **	0.2 *	0	0
Selbach LNA	0	0	0	1 **	0	0
Linsley OME	0.5 *	1 **	1 **	1 **	0	0

Table S12. The number of genes for four gene groups, T -Endo, T +Endo, NT -Endo, and NT -Endo, per experiment

Experiment	T -Endo	T +Endo	NT -Endo	NT +Endo
Grimson	5103	35367	169623	244605
Linsley	1035	57458	222226	300284
Lim	1080	8026	76576	116406
Jackson	1987	12846	318344	500436
Baek	129	728	28992	45934
Selbach	95	1911	48440	75859
Baek MA	834	6444	28287	40218
Selbach MA	766	15518	47769	62252
Selbach LNA	0	280	9707	15274
Linsley OME	0	6388	19414	24720

Table S13. P-values of multiple Wilcoxon rank-sum tests between T -Endo and T +Endo on 5 subgroups of 3' UTR length

Lower p-values indicate that a set of T -Endo genes is more down-regulated than T +Endo genes. “*” and “**” are added when the p-value is less than 0.05 and Sidak adjusted (n=50) 0.001, respectively.

[†] Endogenous miRNAs of the HeLa cells were used to determine if a gene was influenced by any endogenous miRNAs or not (see the Methods). Although some other cell line was used in the Linsley experiment, it is included only for comparison purpose.

[††] Two inhibition experiments, Selbach LNA and Linsley OME, were excluded as they inhibited endogenous miRNAs in the HeLa cells.

Experiment	Very Long	Long		Med Long		Med Short	Short
Grimson	-	0.004507	*	0.9984		0.9912	0.5245
Linsley[†]	-	0.0178	*	0.9859		0.4187	0.6971
Lim	-	3.10E-05	**	0.2057		0.1298	0.1892
Jackson	-	0.7745		0.2359		0.9576	0.3405
Baek	-	0.1639		0.5665		0.7491	0.758
Selbach	-	0.1493		0.005243	*	0.1131	0.3542
Baek MA	-	0.9775		0.915		0.2142	0.8701
Selbach MA	-	0.6107		0.8524		0.7769	0.481
Selbach LNA[††]	-	-		-		-	-
Linsley OME[††]	-	-		-		-	-

Table S14. P-values of multiple Wilcoxon rank-sum tests between T -Endo and T +Endo on 5 subgroups of 3' UTR conservation

Lower p-values indicate that a set of T -Endo genes is more down-regulated than T +Endo genes. “*” and “**” are added when the p-value is less than 0.05 and Sidak adjusted (n=40) 0.0013, respectively.

† Endogenous miRNAs of the HeLa cells were used to determine if a gene was influenced by any endogenous miRNAs or not (see the Methods). Although some other cell line was used in the Linsley experiment, it is included only for comparison purpose.

†† Two inhibition experiments, Selbach LNA and Linsley OME, were excluded as they inhibited endogenous miRNAs in the HeLa cells.

Experiment	High		Medium		Low		NoConsv	
Grimson	0.7221		0.3305		0.6551		0.8161	
Linsley†	0.2087		0.4459		0.3561		0.06864	
Lim	6.97E-07	**	1.29E-07	**	0.003608	*	0.002016	*
Jackson	0.01399	*	0.004478	*	0.0434	*	0.02414	*
Baek	0.6602		0.3803		0.6538		0.2719	
Selbach	0.03193	*	0.01232	*	0.03732	*	0.02471	*
Baek MA	0.2123		0.1387		0.02438	*	0.1335	
Selbach MA	0.2798		0.4313		0.6804		0.5433	
Selbach LNA††	-		-		-		-	
Linsley OME††	-		-		-		-	

Table S15. P-values of multiple Wilcoxon rank-sum tests between T -Endo and T +Endo on 5 subgroups of mRNA expression

Lower p-values indicate that a set of T -Endo genes is more down-regulated than T +Endo genes. “*” and “**” are added when the p-value is less than 0.05 and Sidak adjusted (n=60) 0.00086, respectively.

[†] Endogenous miRNAs of the HeLa cells were used to determine if a gene was influenced by any endogenous miRNAs or not (see the Methods). Although some other cell line was used in the Linsley experiment, it is included only for comparison purpose.

[††] Two inhibition experiments, Selbach LNA and Linsley OME, were excluded as they inhibited endogenous miRNAs in the HeLa cells.

Experiment	Very High	High	Medium	Low	Very Low	NoExp
Grimson	0.2563	0.1928	0.8973	0.6207	0.7154	0.592
Linsley[†]	0.3367	0.6569	3.69E-06 **	0.7857	0.7779	0.6635
Lim	4.81E-07 **	1.98E-08 **	0.0009558 *	0.0001625 **	0.1774	0.5799
Jackson	0.02235 *	0.007227 *	0.0005593 **	0.05376	0.9965	0.1031
Baek	0.5206	0.1892	0.6363	0.7	0.5663	0.9338
Selbach	0.0009269 *	0.003826 *	0.4585	0.3507	0.2129	0.5656
Baek MA	0.03596 *	0.0002753 **	0.4101	0.526	0.1314	0.2536
Selbach MA	0.1056	0.4117	0.04419 *	0.649	0.9644	0.8361
Selbach LNA[††]	-	-	-	-	-	-
Linsley OME[††]	-	-	-	-	-	-

Table S16. Comparisons of Wilcoxon rank-sum tests for T -Endo against T +Endo between experimental and individual sample levels

Expr: the number of experiments that have significant p-value. Smpl: the number of experiments that have Sample level scores greater than either 0.0 (>0.0) or 0.5 (>0.5).

Factor	Subgroup	Expr	Smpl (>0.0)	Smpl (>0.5)
3' UTR Length	Very Long	0	0	0
	Long	3	3	0
	Med Long	1	2	0
	Med Short	0	2	0
	Short	0	3	0
3' UTR Cosv	High	3	2	0
	Medium	3	6	1
	Low	4	4	1
	NoConsv	3	5	0
mRNA Exp	Very High	4	6	0
	High	4	5	1
	Medium	4	4	0
	Low	1	3	0
	Very Low	0	1	0
	NoExp	0	3	0

Table S17. Coefficients and p-values of linear regression with eight factors

Feature	Coef	P-value	-log10p
ln3	-0.01152	4.86E-60	59.31368
cs3	0.009677	3.36E-119	118.4739
exp	0.018357	0	140
#site_m	0.094099	0	140
#endo_m	-0.00423	2.70E-05	4.568619
#site_s	-0.0043	5.06E-31	30.29572
p_ma	0.000495	0.348526	0.457764
e_oe	0.004469	4.56E-37	36.34077

Table S18. Coefficients and p-values of linear regression with eight factors and factor crossing

Feature	Coef	P-value	-log10p
ln3	-2.83E-06	1.22E-05	4.915007242
cs3	-0.015803897	1.18E-05	4.928955818
exp	-0.001124219	0.000303687	3.517574008
#site_m	0.25684395	2.13E-29	28.67115985
#endo_m	0.020685808	0.026462549	1.577368322
#site_s	-0.096087846	0.000458275	3.338874297
p_ma	-0.019081989	0.080483278	1.094294345
e_oe	0.00727338	0.000852402	3.069355424
ln3:cs3	3.94E-07	0.490734868	0.309153084
ln3:exp	-2.84E-07	2.14E-18	17.66958993
ln3:#site_m	1.48E-06	0.176921334	0.752219795
ln3:#endo_m	1.84E-06	1.44E-16	15.84103519
ln3:#site_s	8.68E-07	0.026250076	1.580869438
ln3:p_ma	-4.69E-07	0.401187569	0.396652532
ln3:e_oe	-5.09E-07	0.178249045	0.748972789
cs3:exp	7.59E-05	0.672276785	0.172451885
cs3:#site_m	0.10223823	5.02E-08	7.29904158
cs3:#endo_m	-0.027518299	0.000143836	3.842131652
cs3:#site_s	0.011775068	3.25E-06	5.487463689
cs3:p_ma	0.002299274	0.416319908	0.380572821
cs3:e_oe	0.015830659	1.05E-10	9.977852179
exp:#site_m	0.00899945	1.53E-16	15.81588578
exp:#endo_m	0.000936208	0.041409029	1.382904954
exp:#site_s	-0.000496407	0.002085615	2.680765791
exp:p_ma	0.001429897	2.42E-07	6.61563286
exp:e_oe	0.001102657	2.24E-12	11.64989917
#site_m:#endo_m	-0.221894438	5.61E-45	44.25103531
#site_m:#site_s	0.013159268	0.402069013	0.395699396
#site_m:p_ma	-0.131376751	3.87E-10	9.412586394
#site_m:e_oe	0.073734522	1.98E-10	9.703068939
#endo_m:#site_s	-0.001134625	0.84371691	0.073803247
#endo_m:p_ma	0.000400551	0.95883541	0.018255936
#endo_m:e_oe	-0.008580668	0.145104296	0.838319728
#site_s:p_ma	0.119877581	5.62E-08	7.250318802
#site_s:e_oe	-0.035683116	0.027884011	1.554644753
p_ma:e_oe	0.024313262	0.024118046	1.617657876

Table S19. Coefficients and p-values of linear regression with nine factors

Feature	Coef	Pvalue	-log10p
ln3	-0.00641	3.80E-20	19.42014
cs3	0.004361	1.15E-25	24.93794
exp	0.018288	0	140
#site_m	0.012595	1.04E-06	5.98222
#endo_m	-0.00441	8.56E-06	5.06752
#site_s	-0.00574	1.47E-55	54.83402
p_ma	0.001759	0.000711	3.147907
e_oe	0.004158	2.35E-33	32.62969
ts_score	0.076813	0	140

Table S20. Coefficients and p-values of linear regression with nine factors and factor crossing

Feature	Coef	Pvalue	-log10p
ln3	-1.60E-06	0.011925824	1.923511595
cs3	-0.008366786	0.019318299	1.714031125
exp	-0.001568955	2.59E-07	6.586048885
#site_m	0.159028884	4.01E-10	9.396500422
#endo_m	0.009391765	0.304168389	0.516885922
#site_s	-0.097095613	0.000295034	3.530128515
p_ma	-0.012706529	0.239896623	0.619975865
e_oe	0.003253876	0.128033499	0.892676387
ts_score	0.065575654	7.32E-12	11.13573822
ln3:cs3	9.99E-07	0.079045994	1.102120133
ln3:exp	-2.48E-07	8.53E-15	14.06908328
ln3:#site_m	2.51E-06	0.04108057	1.386363539
ln3:#endo_m	7.29E-07	0.000920571	3.035942721
ln3:#site_s	7.57E-07	0.047937352	1.319325957
ln3:p_ma	-2.36E-07	0.665962223	0.176550406
ln3:e_oe	-1.89E-07	0.609903477	0.214738891
ln3:ts_score	-9.05E-06	8.39E-19	18.07603485
cs3:exp	-0.000210957	0.234127943	0.630546751
cs3:#site_m	0.063088756	0.003621569	2.44110321
cs3:#endo_m	-0.030869619	1.57E-05	4.804938386
cs3:#site_s	0.011989538	1.74E-06	5.758989552
cs3:p_ma	-0.000515518	0.853673874	0.06870801
cs3:e_oe	0.011256547	3.69E-06	5.433102786
cs3:ts_score	-0.056098519	1.49E-14	13.82670741
exp:#site_m	0.002073852	0.075893396	1.119796014
exp:#endo_m	0.001163854	0.009384581	2.027585129
exp:#site_s	-0.000576086	0.000255828	3.592052003
exp:p_ma	0.001497452	3.07E-08	7.512962288
exp:e_oe	0.001052792	6.52E-12	11.18585865
exp:ts_score	0.008284526	3.99E-66	65.39935915
#site_m:#endo_m	-0.050965831	0.001854836	2.731694567
#site_m:#site_s	0.079648242	1.72E-06	5.764690568
#site_m:p_ma	-0.182652224	8.11E-15	14.0911118
#site_m:e_oe	0.011835961	0.335633255	0.474135015
#site_m:ts_score	-0.016918648	0.320827576	0.49372831
#endo_m:#site_s	-0.00650073	0.248378993	0.604885138
#endo_m:p_ma	0.003415606	0.652117952	0.185673844
#endo_m:e_oe	-0.008238802	0.151727348	0.818936132
#endo_m:ts_score	0.044847948	0.00152743	2.8160388
#site_s:p_ma	0.110263074	2.75E-07	6.561220289
#site_s:e_oe	-0.027099317	0.091634949	1.037938855
#site_s:ts_score	-0.035149695	8.23E-08	7.084730455
p_ma:e_oe	0.019092069	0.073869883	1.13153259
p_ma:ts_score	0.04113798	8.56E-07	6.067689011
e_oe:ts_score	0.06617109	2.56E-33	32.59133846

Table S21. P-values of multiple Wilcoxon rank-sum tests on 3 subgroups of CpG frequency

Lower p-values indicate that a set of genes in one sub-group is more down-regulated than a set of genes in the rest of subgroups. "*" and "**" are added when the p-value is less than 0.05 and Sidak adjusted (n=30) 0.0017, respectively.

Experiment	High		Med		Low
Grimson	4.84E-27	**	1.60E-05	**	1
Linsley	1.44E-115	**	0.8724		1
Lim	0.001451	**	0.3292		1
Jackson	4.49E-14	**	0.01002	*	1
Baek	0.1548		0.9116		0.283
Selbach	0.7002		0.1597		0.8271
Baek MA	0.005732	*	0.04945	*	1
Selbach MA	0.001856	*	0.0005601	**	1
Selbach LNA	0.4183		0.6502		0.3786
Linsley OME	5.89E-08	**	0.8315		1

Table S22 P-values of multiple Wilcoxon rank-sum tests on 2 subgroups of developmental genes

Lower p-values indicate that a set of genes in one sub-group is more down-regulated than a set of genes in the rest of subgroups. "*" and "**" are added when the p-value is less than 0.05 and Sidak adjusted (n=20) 0.0026, respectively.

Experiment	Dev		NonDev	
Grimson	0.9961		0.003889	*
Linsley	0.9917		0.008313	*
Lim	0.966		0.03401	*
Jackson	0.9988		0.001185	**
Baek	0.05068		0.9494	
Selbach	0.09926		0.9008	
Baek MA	0.7844		0.2156	
Selbach MA	2.74E-05	**	1	
Selbach LNA	0.4256		0.5751	
Linsley OME	0.8274		0.1726	

Table S23. P-values of multiple Wilcoxon rank-sum tests on 2 subgroups of housekeeping genes

Lower p-values indicate that a set of genes in one sub-group is more down-regulated than a set of genes in the rest of subgroups. "*" and "**" are added when the p-value is less than 0.05 and Sidak adjusted (n=20) 0.0026, respectively.

Experiment	HK		NonHK	
Grimson	2.93E-06	**	1	
Linsley	1.67E-08	**	1	
Lim	0.0001003	**	0.9999	
Jackson	1.62E-06	**	1	
Baek	0.7208		0.2794	
Selbach	0.876		0.1241	
Baek MA	0.07838		0.9216	
Selbach MA	0.09997		0.9	
Selbach LNA	0.1272		0.8733	
Linsley OME	0.9771		0.02288	*

Supplementary Figures

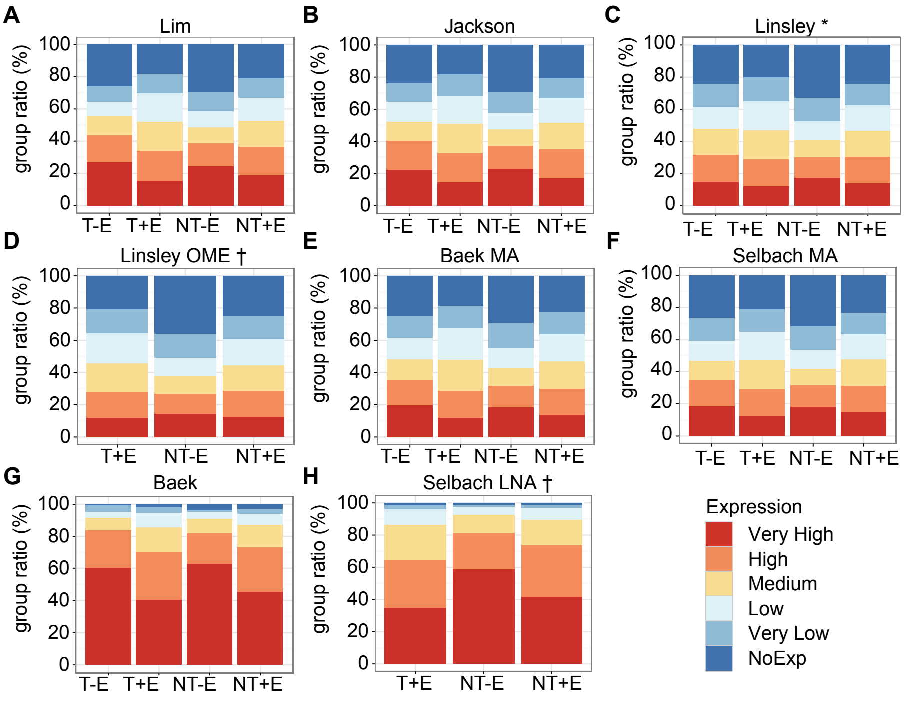

Figure S1. mRNA expression levels from 6 microarray and 2 proteomics experiments. We calculated the ratios of tag counts of RNASeq for each sub-group relative to the total counts for 6 microarray (A-G) and 2 proteomics (G and H) experiments. The experiment data were divided into four by exogenous miRNA targets (T) and non-targets (NT), as well as endogenous miRNA targets (+E) and non-targets (-E).

* Endogenous miRNAs of the HeLa cells were used to determine if a gene was influenced by any endogenous miRNAs or not (see the Methods). Although some other cell line was used in the Linsley experiment, it is included only for comparison purpose.

† As two inhibition experiments, Selbach LNA and Linsley OME, inhibited endogenous miRNAs in the HeLa cells, there were no genes categorized as T -Endo.

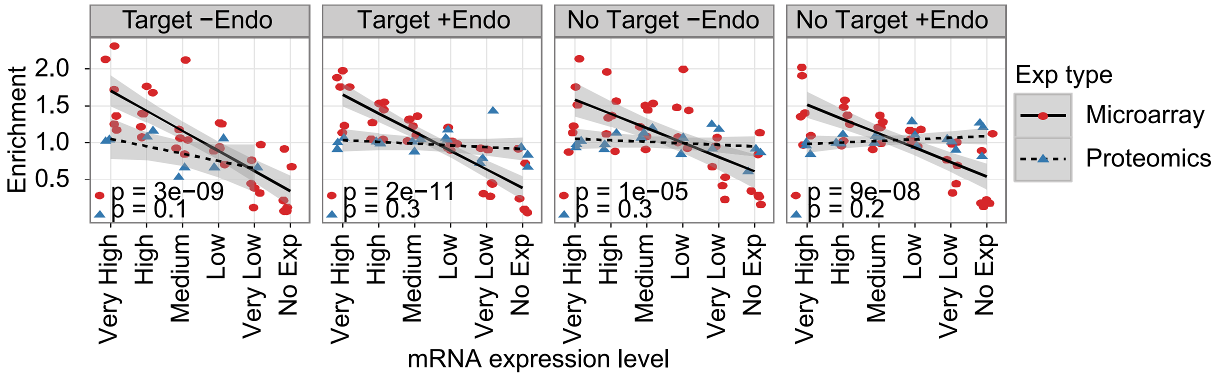

Figure S2. Log2 enrichment of down-regulated genes compared with all genes mRNA. Scatter plots show log2 enrichment of down-regulated genes compared with all genes for the six sub-groups of mRNA expression levels in all studied datasets subdivided by predicted exogenous and endogenous miRNA targeting. Lines and shaded grays show respectively linear fits and standard errors for the microarray (red dots) and proteomics (blue triangles) experiments; p-values (lower left) are unadjusted p-values from Pearson correlation tests. Data points based on a single gene were excluded. The regression lines show that in the microarray but not the proteomics experiments, down-regulated genes are enriched among highly expressed genes and that this enrichment depends on gene expression levels.

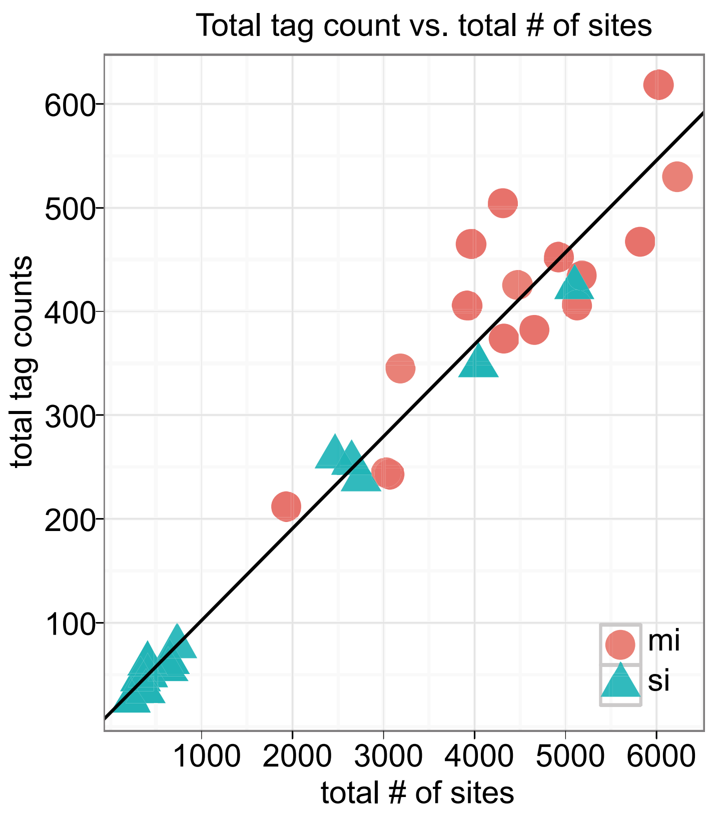

Figure S3. Scatter plot of the total tag counts versus total number of miRNA target sites. We plotted a scatter plots to check the correlation between mRNA expression level and the number of miRNA target sites. The plot shows the total tag counts and the total number of miRNA target sites from 67 samples with 32 miRNAs and 35 siRNAs. The line was drawn by Pearson's correlation (r = 0.973 and p 2.2e-16). Only the samples assayed in HeLa were included. Red circles represent miRNAs, whereas blue triangles represent siRNAs.

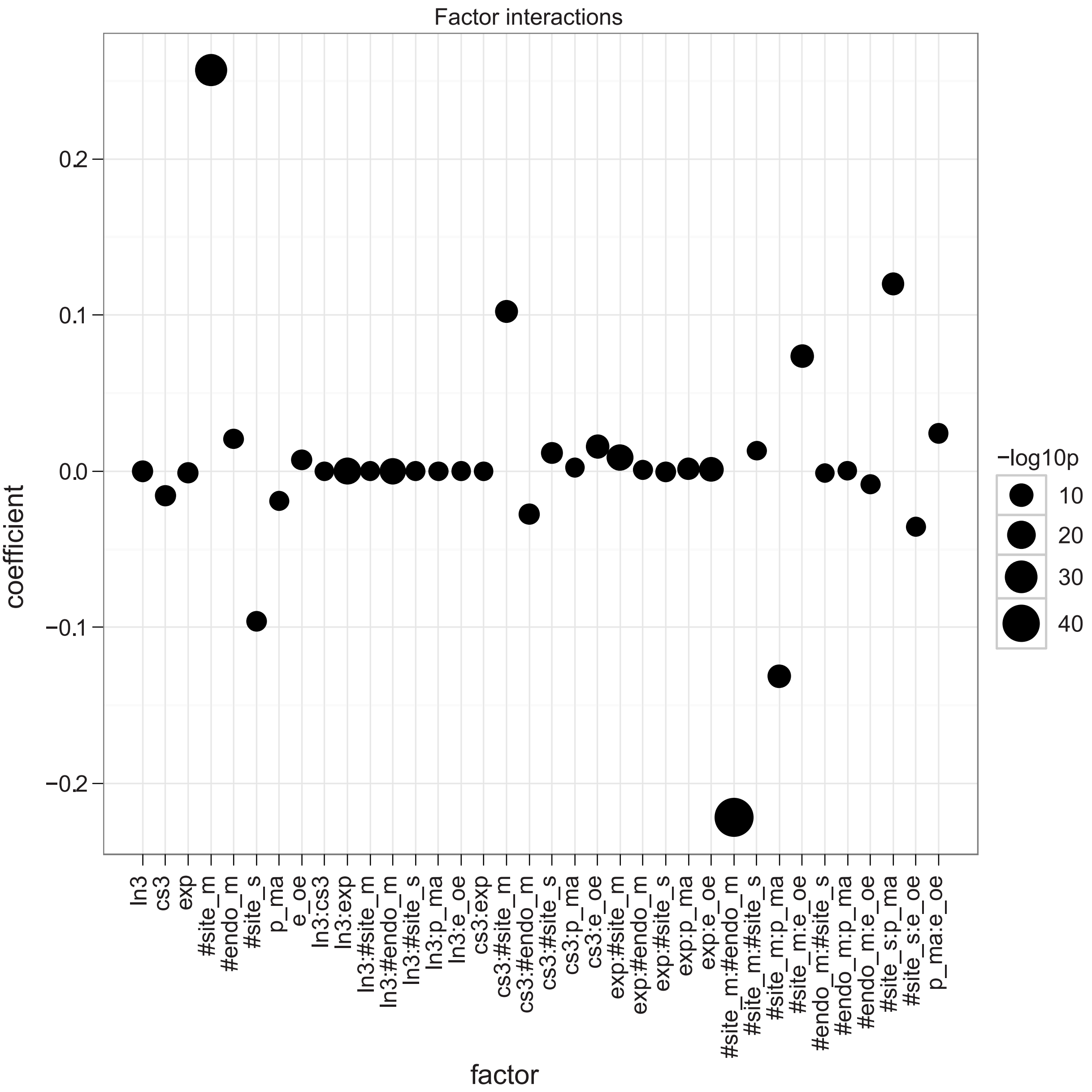

Figure S4. Coefficients of a linear regression with eight factors and factor crossing

The dot plot shows the coefficients of the liner model with formula: -logratio = (ln3 + cs3 + exp + #site_m + #endo_m + #site_s + p_ma + e_oe)^2. The ^ operator extracts all eight factors as well as all possible combinations of second order interactions, such as ln3 * cs3 (ln3:cs3), ln3 * exp (ln3:exp), and so on. The size of dots indicates -$\log_{10}$p as the negation of the logarithm of p-values to base 10. Positive coefficients associate with miRNA down-regulation.

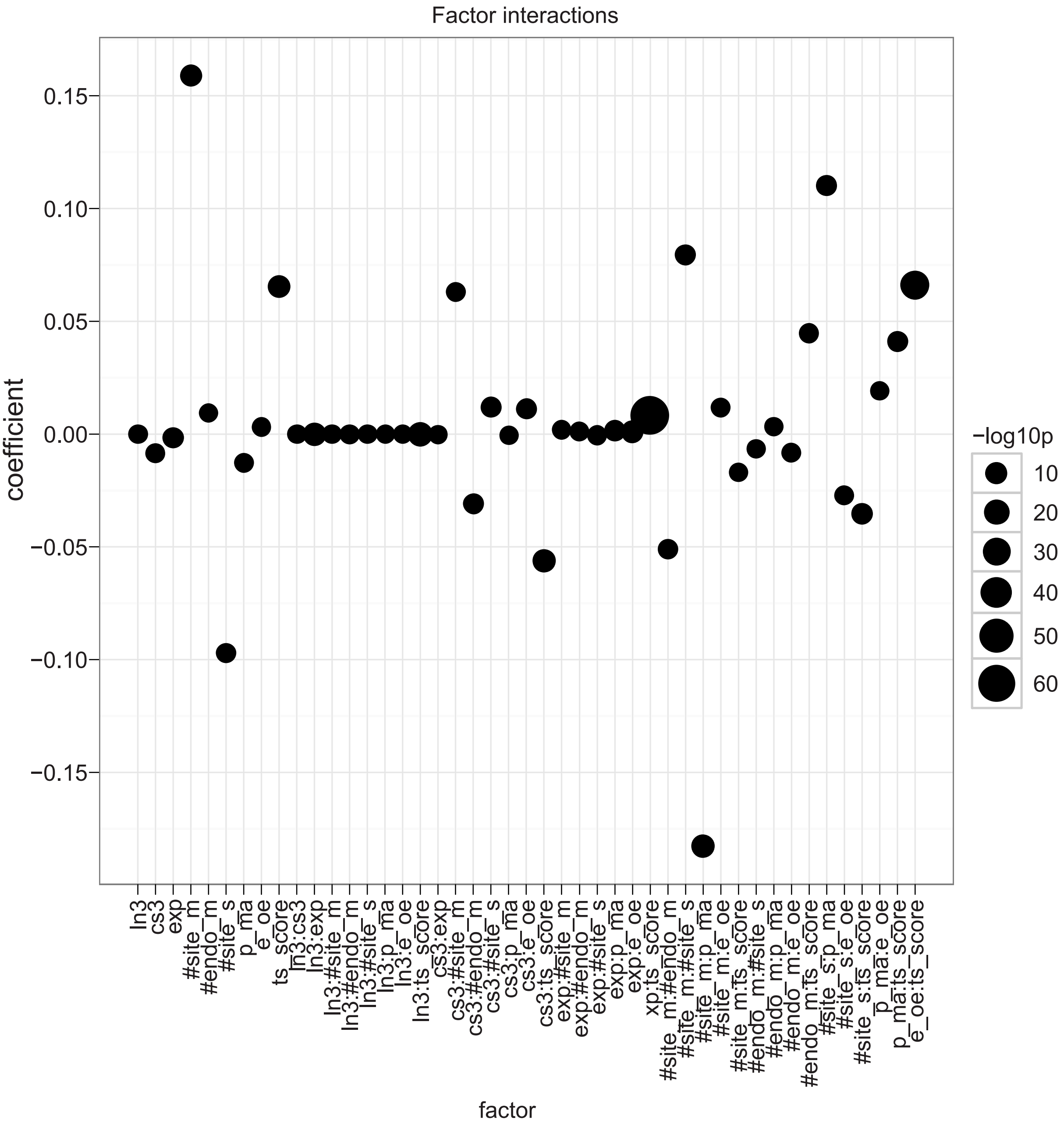

Figure S5. Coefficients of a linear regression with nine factors and factor crossing
The dot plot shows the coefficients of the liner model with formula: -logratio = (ln3 + cs3 + exp + #site_m + #endo_m + #site_s + p_ma + e_oe + ts_score)^2. The ^ operator extracts all eight factors as well as all possible combinations of second order interactions, such as ln3 * cs3 (ln3:cs3), ln3 * exp (ln3:exp), and so on. The size of dots indicates -$\log_{10}$p as the negation of the logarithm of p-values to base 10. Positive coefficients associate with miRNA down-regulation.
